# Alkali metal cation effects for rapid C–H activation by iron(0) complexes

**DOI:** 10.1039/d6sc01470d

**Published:** 2026-03-31

**Authors:** Nereida Hidalgo, Kendal W. Southwell, Ryan S. Donnelly, Brandon Q. Mercado, Sebastian M. Krajewski, Xiaoping Wang, Patrick L. Holland

**Affiliations:** a Department of Chemistry, Yale University New Haven CT 06520 USA patrick.holland@yale.edu; b Neutron Scattering Division, Oak Ridge National Laboratory Oak Ridge TN 37831 USA xwang@ornl.gov

## Abstract

Ion pairing with alkali metal cations offers a novel way to enhance the rate of C–H activation. This strategy is demonstrated here using diketiminate-supported iron(0) complexes, which previously were shown to activate the C–H bond of benzene slowly (hours at room temperature) and did not reach completion before decomposition. However, by removing a crown ether that sequesters the alkali metal countercation, there is rapid C–H oxidative addition in less than a minute at ambient temperature, showing that alkali cation effects can strongly influence C–H activation, even with the more weakly interacting Rb and Cs. The products from C–H activation are unusual dimeric iron(ii) phenyl hydrides bridged by K^+^, Rb^+^, or Cs^+^ cations. Because locating bridging hydrides in the middle of heavy atoms is challenging, neutron diffraction was used to conclusively locate the bridging hydride atoms for the Cs derivative. These results establish “alkali control” as a method for tuning the rate and outcome of C–H activation reactions by anionic iron complexes.

## Abstract 2

Ion pairing with alkali metal cations offers a novel way to enhance the rate of C–H activation.

## Graphical abstract

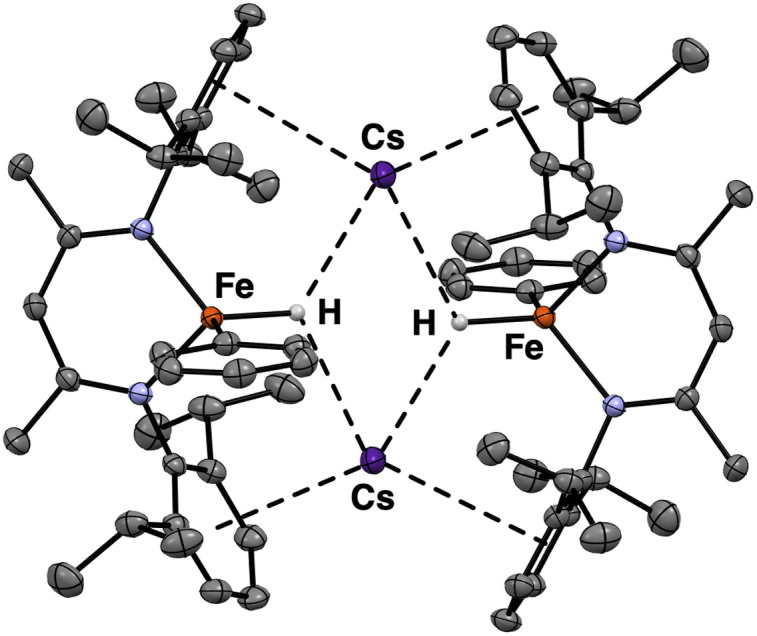

## Introduction

The cleavage and functionalization of otherwise inert carbon-hydrogen (C–H) bonds has become a powerful tactic in modern synthesis.^1–4^ C–H activation frequently minimizes the number of synthetic steps required for building complex molecules.^5–7^ Though most C–H activation research has used precious metals, the current drive toward more sustainable catalysis has shifted attention toward abundant base metals like iron.^8–11^

**Scheme 1:**
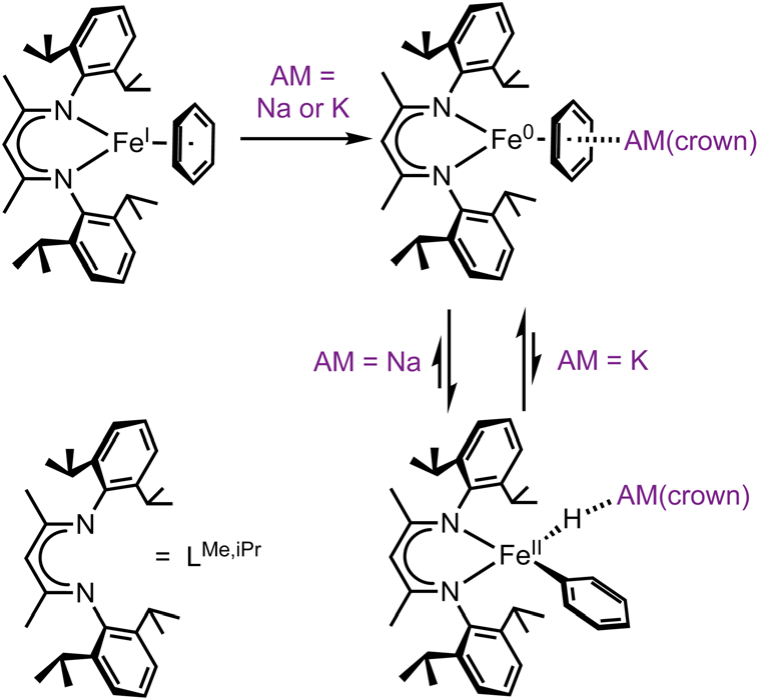
Iron(0) complexes perform C–H activation, dependent on the alkali metal (AM) cation. Sodium favors the iron(ii) oxidative addition product, while potassium favors the iron(0) species.

Several mechanistic pathways for iron-mediated C–H activation have been explored.^12–15^ Here we focus on C–H oxidative addition by iron(0) compounds to give organometallic iron(ii) hydride species, which is relevant to a number of stoichiometric and catalytic reactions.^16–23^ In our research, the exploration of iron(0) organometallic chemistry began with the reaction of the iron(i)-benzene complex L^Me,iPr^Fe(C_6_H_6_)^24^ with the reductant KC_8_ in the presence of 18-crown-6, which gave an isolable *η*^6^-benzene complex of iron(0) that has a high-spin electronic configuration.^25^ When the reduction was done instead with sodium sand and 15-crown-5, the product was instead a paramagnetic phenyl hydride complex of iron(ii) (Scheme 1).^26^ This implied that there had been oxidative addition of a benzene C–H bond, and we further showed that the resulting iron phenyl intermediates could activate N_2_ to eventually give C–N bond formation and aniline derivatives through a one-pot route.^26^ This synthetic cycle to aminate benzene with N_2_ could in principle become catalytic if the yields and rates of the individual steps could be improved. A key barrier was slow C–H activation, which required 1–2 hours.

C–H activation by the iron(0) intermediate was dependent on the alkali metal (AM) used in its preparation.^26^ As noted above, sodium/15-crown-5 led to slow oxidative addition, while potassium or rubidium reductants in the presence of 18-crown-6 left the iron(0) arene complex without oxidative addition. Thus, the alkali metal had a drastic influence on the thermodynamics of the C–H oxidative addition/reductive elimination (OA/RE) equilibrium. The reason for this influence was not initially clear, as AM cations can play various roles such as pre-organizing metal centers, creation of electric fields, and bonding to Lewis bases.^27^ We anticipated a broad impact from developing an understanding of this phenomenon, because AM cations affect other iron-based C–H activations as well. For example, studies by Lefèvre show that AM salts of hexamethydisilazide can be used as either bases or reductants with different C–H substrates in iron halide systems depending on the nature of the alkali cation.^22^ In addition, Hevia has shown that iron and alkali metals may be paired for other kinds of catalytic reactivity.^28^ Controlling the coordination environment of alkali metal cations, whether by varying the cation itself or modifying its encapsulation, offers a powerful yet underexplored method for modulating the rate and selectivity of iron-mediated C–H activation reactions.

For insight into the thermodynamic and kinetic determinants of alkali control in the β-diketiminate system, we pursued DFT calculations.^29^ Energy decomposition analysis showed that Na(15-crown-5) favored the iron(ii) phenyl hydride isomer due to a closer electrostatic interaction, while K(18-crown-6) and Rb(18-crown-6) favored the arene-bound iron(0) isomer because of steric repulsion and the reorganization required to bind larger crown ethers. The changes in the heights of the barriers for OA/RE were also explored computationally. These computations revealed a lower barrier with Na(15-crown-5), and a higher barrier with K(18-crown-6) or Rb(18-crown-6), with the difference attributed to steric effects. Computations on unsolvated AM^+^ contact ion pairs, on the other hand, suggested that the C–H activation barrier is lower in the absence of a crown ether. These inspired the experimental studies below, which show that omitting crown ether indeed leads to rapid benzene C–H oxidative addition at room temperature, consistent with a stronger alkali metal effect in the contact-ion-paired system.

A recurrent challenge with C–H activation reactions is that the product has a hydride, but hydrides are notoriously difficult to conclusively identify in paramagnetic compounds.^30^ The putative hydride formed in the oxidative addition reaction described above is particularly challenging because it is surrounded by heavy atoms, which create Fourier “ripples” that can mask the small electron density from the hydride. Therefore we use neutron diffraction, as the distinctive negative scattering length for ^1^H nuclei yields definitive identification and location of hydrides in the core of the molecule.

## Results and discussion

### Formation and structures of phenyl hydride complexes from C–H activation

The reduction reactions of the red iron(i) benzene complex L^Me,iPr^Fe(C_6_H_6_)^24^ with alkali metal graphite reagents KC_8_, RbC_8_, or CsC_8_ under an Ar atmosphere each resulted in an instantaneous color change to deep purple. In each case ^1^H NMR spectra in C_6_D_6_ at room temperature recorded after approximately 10 min showed 4–5 paramagnetically shifted peaks (SI, Fig. S1–S3), consistent with rapid conversion to a major product. These products are labeled 1 for K, 2 for Rb, and 3 for Cs. Crystals of each were obtained from concentrated toluene solutions at −40 °C. It was essential to analyze these samples soon after crystallization, as storage for more than a day even at −40 °C in the glove box led to decomposition.

**Fig. 1:**
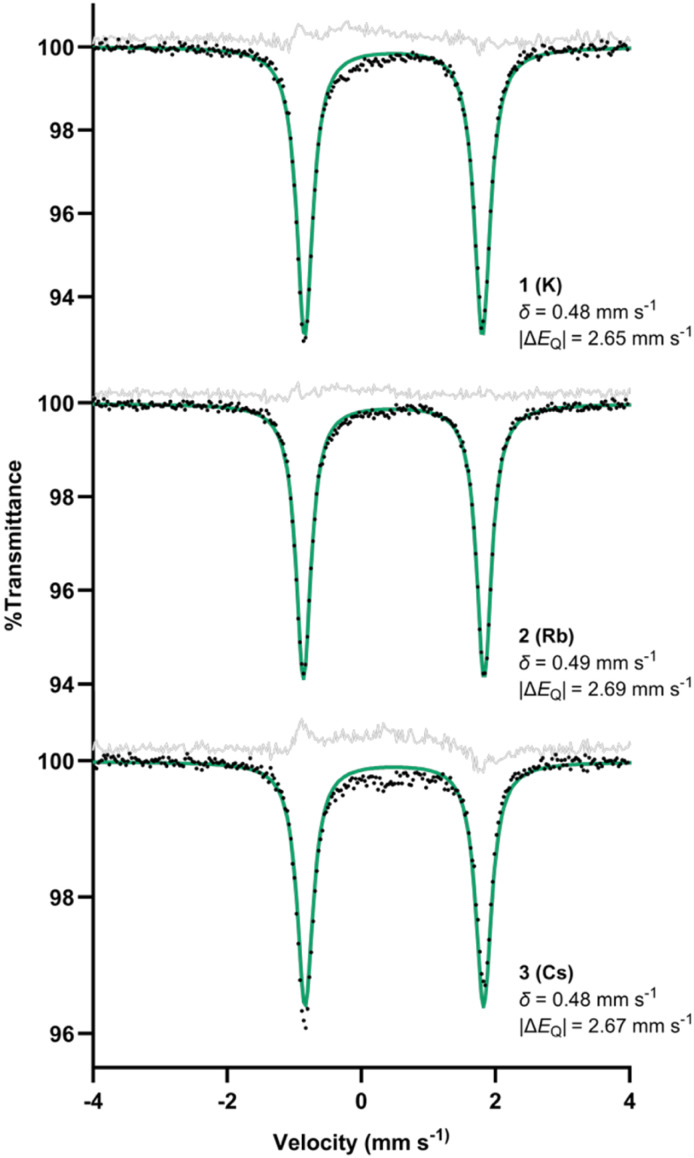
Zero-field Mössbauer spectra of solid 1, 2, and 3 at 80 K. Alternative fits of 3 incorporating a 10–15% impurity are given in the SI (Fig. S37 and S38).

Low stability and the potential for impurities hindered spectroscopic characterization in solution (*vide infra*). Solid-state measurements required less manipulation, and thus are more reliable. The solid-state Mössbauer spectrum of each new complex at 80 K showed a quadrupole doublet with an isomer shift (*δ*) of 0.48–0.49 mm s^−1^ (Fig. 1). These values are similar to the isomer shift (0.54 mm s^−1^) previously observed for the monomeric iron(ii) phenyl hydride with sodium and 15-crown-5.^26^ They are also similar to the isomer shifts for other β-diketiminate-supported diiron(ii) complexes with a hydride bridge and an aryl ligand (0.53 and 0.59 mm s^−1^).^31^ The quadrupole splittings are 2.65–2.69 mm s^−1^, and such large splitting values are typical for high-spin (*S* = 2) iron(ii) compounds.^32^

**Scheme 2:**
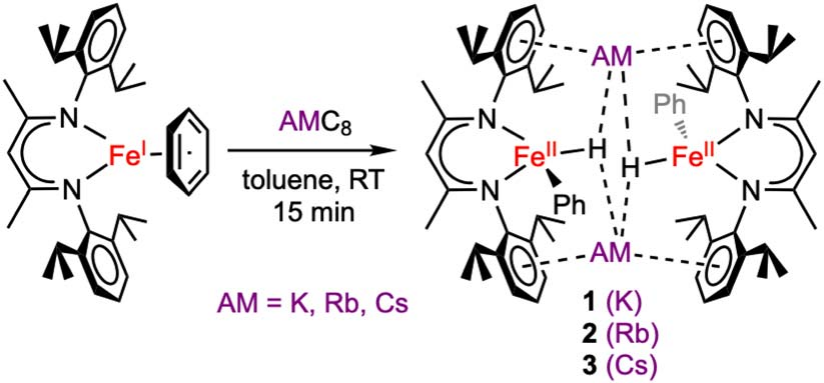
Formation of metastable iron(ii) phenyl hydride complexes, which are dimers bridged by alkali metal (AM) cations.

**Fig. 2:**
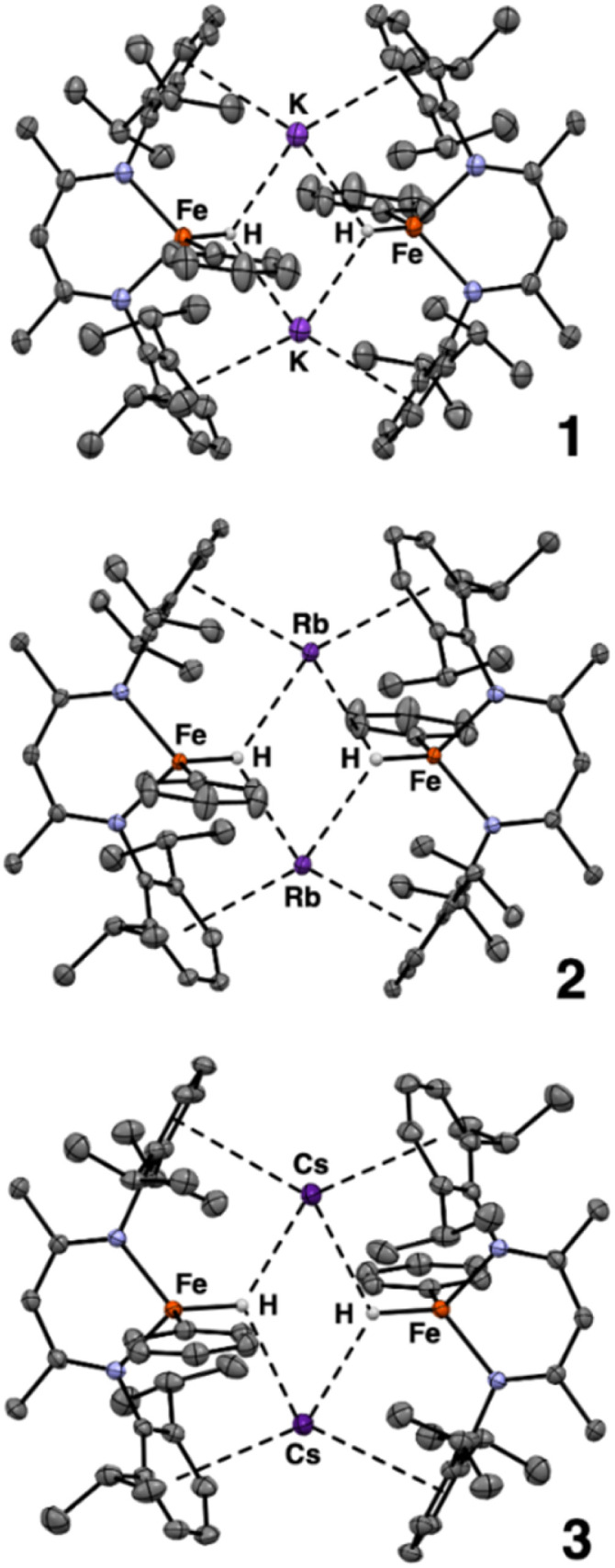
ORTEP plots of the X-ray crystal structures of 1, 2, and 3 using 50% thermal ellipsoids. The pseudo-*C*_2_ axes are vertical in this view. Hydrogen atoms other than hydrides are not shown for clarity.

Using single crystals, we performed X-ray diffraction (XRD) analysis of each new product. All three are dimeric iron(ii) species in which two β-diketiminate-supported Fe units are bridged by alkali metal cations (Scheme 2). The structures of 1–3 are closely analogous to one another, and each has idealized *C*_2h_ symmetry arising from a crystallographic inversion center with a pseudo-mirror plane bisecting each β-diketiminate (Fig. 2). Each Fe atom has one phenyl group, projecting from a different side of the core. The angles between the Fe–C bonds and the best-fit (β-diketiminate)Fe planes (109–115°, see Table 1) suggest that the two N and one C donor are not the only ligands. Instead, the geometry is consistent with a tetrahedral Fe center bearing an aryl ligand and a putative hydride ligand, a hypothesis tested below using neutron crystallography on 3. Close AM⋯π interactions (AM = K, Rb, Cs) to the aromatic substituents of the β-diketiminate ligands (3.1–3.4 Å) are notable and resemble those previously described for a dimeric iron(i) hydride complex with K^+^ countercations incorporated into the structure,^33^ and for bridging N_2_ complexes with K, Rb, and Cs connecting the aromatic rings.^34^

**Table 1:** Key metrical parameters in 1–3

	1(K)	2(Rb)	3(Cs)
Fe–N (Å)	2.030(5)	2.010(3)	2.019(2)
	2.014(4)	2.027(4)	2.024(2)
Fe–C (Å)	2.064(6)	2.065(5)	2.066(2)
AM–centroid of arene (Å)	3.10	3.19	3.41
	3.14	3.17	3.37
C(L^Me,iPr^)–Fe–C(aryl) (°)	115	109	115

### Neutron crystallography of a dimeric hydride complex

As noted above, the sharp angle to the phenyl group is consistent with the presence of a hydride ligand, and the Fourier maps from the X-ray crystal structures show a peak located between Fe and the alkali-metal cations consistent with a bridging hydride. However, hydride assignment by X-ray diffraction is intrinsically uncertain, because X-rays probe electron density. Since the single electron on hydrogen is involved in bonding it is often hard to detect, and its apparent position is typically displaced toward the heavier atom. In addition, limited resolution and the proximity of heavy atoms (Fe and Cs) can also generate “ripples” in the electron-density map that mimic or obscure hydrogen positions. Therefore, we turned to single-crystal neutron diffraction on 3 to obtain an unambiguous identification of the hydride and its location. Neutrons are scattered by nuclei, and therefore do not suffer from the aforementioned issues. Notably, the coherent scattering length of ^1^H is substantial and negative (−3.74 fm), whereas C, N, Fe and Cs have positive scattering lengths.^35^ This sign contrast yields a distinctive negative nuclear density feature in Fourier difference maps, enabling reliable discrimination of hydride from other atoms.

**Fig. 3:**
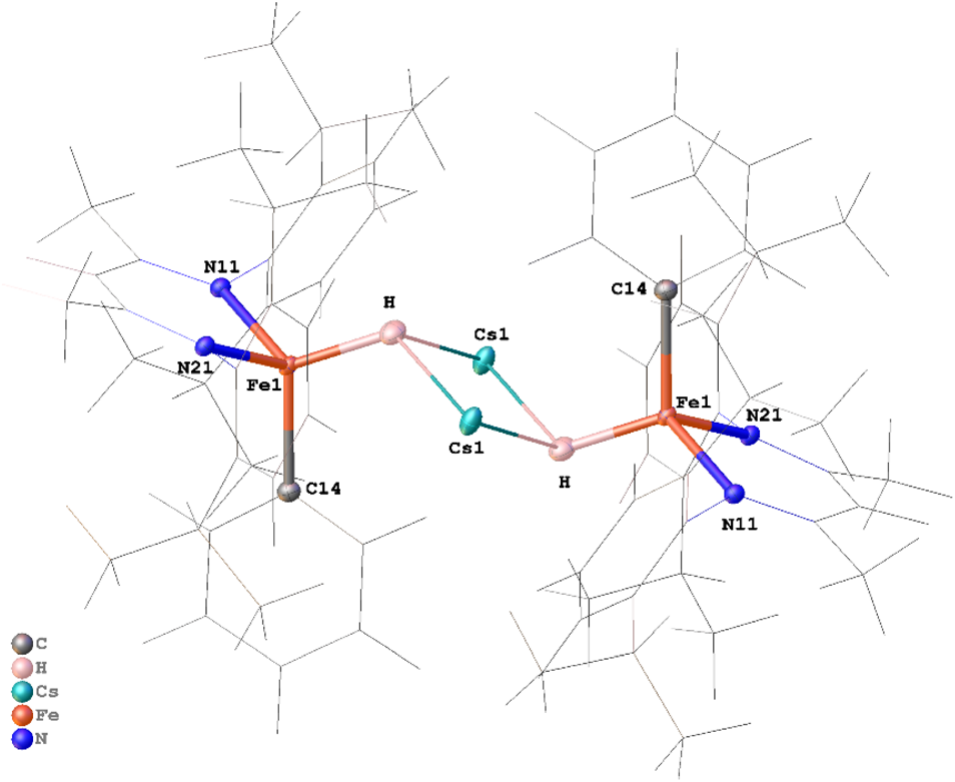
ORTEP plot of the neutron crystal structure of 3, with 30% thermal ellipsoids on the Cs_2_Fe_2_H_2_ core. Atoms of the ligand framework not directly bonded to Fe are shown in wireframe for clarity. Selected distances (Å) and angles (°): Fe_1_–H 1.71 (5), Cs_1_–H 3.04 (5), Cs_1_–H^i^ 2.92 (4); Fe_1_–H–Cs_1_ 102.4 (18), Fe_1_–H–Cs_1_^i^ 114 (2), Cs_1_^i^–H–Cs_1_ 115.6 (14), H^i^–Cs_1_–H 64.4 (14). *i* indicates symmetry-equivalent atoms at positions −*x* + 1, −*y* + 1, −*z* + 1.

Despite the plate-like habit and small volume of the crystal (0.36 × 0.25 × 0.05 mm; *ca.* 0.005 mm^3^), 874 independent neutron reflections were indexed at the TOPAZ beamline at the Spallation Neutron Source, Oak Ridge National Laboratory.^36^ Because the neutron dataset was too small for a conventional full-matrix refinement of all positional and anisotropic displacement parameters, we exploited the complementarity of X-ray and neutron data using Hirshfeld atom refinement (HAR), as implemented in NoSpherA2,^37^ on the high-quality X-ray data to define the heavy atom framework, and then refined only the hydride position against the neutron intensities. The X-ray dataset was first refined against a DFT-derived electron-density model (PBE/jorge-DZP-DKH with DKH2 relativistic treatment),^38^ yielding an accurate description of the heavy-atom framework, including non-spherical electron density in the Fe–N, Fe–C, and Cs–π bonding regions. In the second step, all non-hydrogen atoms were fixed at their HAR positions and ADPs, C-bound hydrogen atoms were treated with riding models, and only the putative hydride was refined against the neutron data. This procedure produced a well-defined negative nuclear density peak between Fe and Cs that could be refined with reasonable anisotropic displacement parameters as shown in the ORTEP diagram in Fig. 3. To minimize model bias in the hydride assignment, we also calculated an *F*_o_ − *F*_c_ neutron omit map with the bridging hydride removed from the model (Fig. S33). The map shows a well-defined negative nuclear density feature at the same position. The resulting Fe–H distance of 1.71(5) Å agrees with the HAR Fe–H distance of 1.77(2) Å from the X-ray analysis, providing strong, independent evidence for a genuine hydride ligand at this position.

The hydride engages not only with the Fe atoms: in the X-ray model, Cs–H distances of 2.97(2) and 2.96(2) Å, together with Fe–H–Cs angles of 103.2(9)° and 110.3(9)°, define a Cs_2_Fe_2_H_2_ core (Fig. 3). The core is reminiscent of that in a reported β-diketiminate supported diiron(i) hydride complex^33^

### Rapid C–H activation

It is notable that the C–H activation of benzene that leads to 1–3 occurs in seconds. This contrasts with the >1 hour required for C–H activation using Na in the presence of crown ether.^26^ This difference demonstrates that the alkali cation is involved in the transition state for C–H activation. These observations are consistent with the idea that removing the crown ether increases the effective Lewis acidity and accessibility of the alkali metal cation. The crown-free alkali metal cation can then more effectively lower the barrier for C–H oxidative addition.

The thermodynamics also change: with crown ethers, the iron(0) benzene isomer was favored over the iron(ii) phenyl hydride isomers for K, Rb, and Cs,^26,29^ whereas in the crown-free system described here, the C–H activated product predominates with these heavier alkali metal cations. It is not possible from our studies to distinguish whether this results solely from changes in the Lewis acidity of the crown-free alkali metal cation, or from the dimerization (well-known to hold previous dimeric β-diketiminate complexes together).^39^ Considering the computational evidence for greater Lewis acidity,^29^ we favor this explanation for the thermodynamics as well.

Several additional observations are worth noting. Varying the reductant stoichiometry using excess KC_8_, RbC_8_ or CsC_8_ (up to 3 equiv.) did not alter the ^1^H NMR spectra or product distribution (SI, Fig. S7–S9), suggesting that further reduction does not occur under these conditions. Also, exposure of solutions of 1, 2, or 3 to N_2_ results in new signals in ^1^H NMR spectra more quickly than the decomposition under Ar, but the paramagnetic product(s) could not be identified. Future studies will evaluate the interesting prospects that arise from their rapid reactions with N_2_.

### Reactivity of the C–H activation products with crown ethers and cryptands

The coordination sphere of the AM^+^ ion is intimately involved in the assembly and reactivity of dimeric iron complexes.^39^ To explore the effect of chelating the AM^+^ after construction of the dimer, we treated 1–3 with 2 equiv. (1 per Fe) of 18-crown-6 in toluene-*d*_8_ at −78 °C and monitored the solutions using ^1^H NMR spectroscopy (SI, Fig. S13–S15). The initial spectra below −50 °C closely resemble the monomeric sodium analogue,^26^ and we conclude that the dimers break up into iron(ii) phenyl hydride monomers. In one case, we were even able to obtain a single crystal whose diffraction data indicated the composition [Cs(18-crown-6)_2_][L^Me,iPr^Fe(Ph)(H)], supporting this idea (see SI Fig. S36).

Warming these solutions to room temperature gave ^1^H NMR spectra that indicate degradation with an onset around −20 °C. The broadness of the spectra, and the shifting due to paramagnetism, hindered our attempts to quantify the degradation rates, but the changes clearly indicate that the monomeric iron(ii) phenyl hydride complexes are very unstable in solution when they have crown on K/Rb/Cs instead of the Na(15-crown-5) in the earlier compound.^26^

**Scheme 3:**
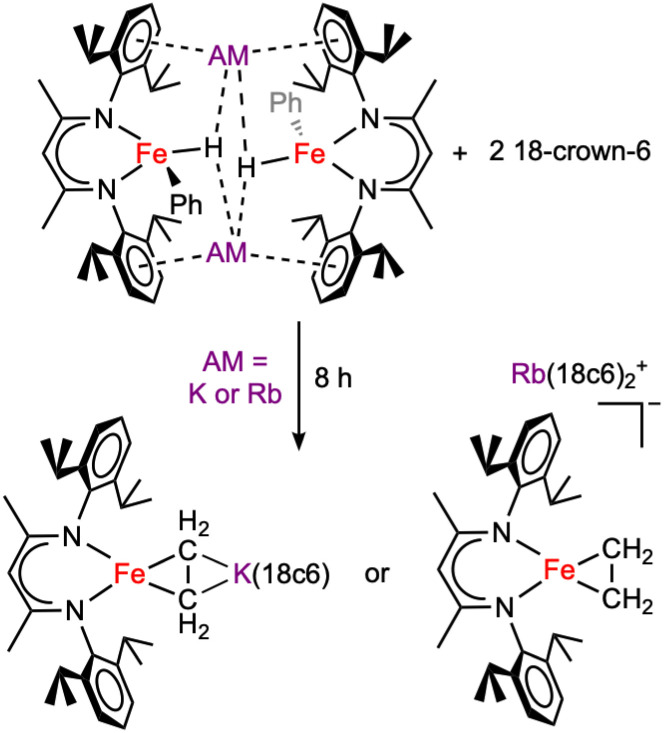
Decomposition of 1 and 2 through crown ether cleavage. These products are not fully characterized, and yields are not known, but were identified using crystallography.

The nature of most degradation products is not clear. However, some samples of 1 or 2 that stood for *ca.* 8 h at −35 °C in toluene gave crystalline material suitable for X-ray diffraction. Although we did not quantify the yields or further characterize these products, the crystal structures show formally iron(0) ethylene complexes (Scheme 3, and SI Fig. S34–S35). It is likely that the ethylene arises from cleavage of the 18-crown-6 macrocycle under the reaction conditions, as examples of reductive C–O bond cleavage of crown ethers are documented.^40,41^

In addition to the crown ethers, we also sought a chelator that would completely encapsulate the metal cation. Treating 1–3 with 2.2.2-cryptand under argon gave immediate degradation as judged by ^1^H NMR spectroscopy (SI, Fig. S16–S19). This suggests that complete sequestration of AM^+^ by the cryptand unmasks a very reactive and unstable fragment. This is reminiscent of a system in which addition of a cryptand to a dimeric aluminyl complex resulted in cleavage of a benzene C–C bond.^42^

## Conclusion

With crown ether present, larger alkali metals like K, Rb, and Cs gave an iron(0) benzene complex without C–H activation, whereas the crown-free system here gives complete oxidative addition with these metals.^26,28^ In the presence of crown, complete oxidative addition had only occurred with more Lewis acidic Na, but here we show that omitting crown enables the less Lewis acidic K, Rb, and Cs to drive the equilibrium. Taken together, these trends suggest that crown ether binding reduces the effective Lewis acidity and accessibility of the alkali metal cation to the point that only the higher charge density of Na^+^ can overcome this moderating effect, whereas without crown ether even larger AM^+^ can participate strongly enough to favor oxidative addition.

With respect to the rates, the removal of crown renders C–H oxidative addition rapid with all alkali metals, consistent with the hypothesis^29^ that steric effects from the crown ether can raise the energy of the transition state for C–H activation. It is significant that the omission of the crown ether makes the effect of the alkali metal cation more marked, a lesson that may have more general applicability for alkali metal cation effects in catalysis. Though coordination chemists often add a crown ether to dissolve the alkali metal cation, our results show that the crown can alter both outcomes and rates in reactions where the alkali metal cation participates. In the future, we plan to apply these principles to the alkali-cation-promoted coupling of benzene and N_2_ to generate anilines.^26^

The products of C–H activation are dimers held together by AM^+^, which binds to both the hydrides and the π electrons of the β-diketiminate aryl groups. These AM^+^ interactions with the bridging hydrides may rigidify the core and stabilize the C–H activation products. This idea is reinforced by the observation that treatment with crown ethers or cryptands triggers rapid degradation, including destruction of the crown ether.

To our knowledge, the only previous examples of AM^+^ control over C–H activation occurred in a concerted metallation-deprotonation mechanism (where AM^+^ interacted with an accessory acetate ligand),^43^ and in C–H deprotonation reactions (where AM^+^ is part of a strong base).^22,28^ AM^+^ control over C–H oxidative addition is previously unknown to our knowledge. We propose that in anionic transition-metal complexes and relatively nonpolar solvents, where ion pairing is important, the ability of AM^+^ to interact with arenes and with hydrides has potential to induce rate and/or selectivity effects on C–H oxidative reaction mechanisms.

## Author contributions

Conceptualization: N. H., P. L. H.; formal analysis and investigation: N. H., K. W. S., R. S. D., B. Q. M., S. M. K., X. W.; supervision: X. W., P. L. H.; writing – original draft: N. H., X. W.; writing – review & editing: N. H., K. W. S., X. W., P. L. H.

## Conflicts of interest

There are no conflicts to declare.

## Supplementary Material

SC-OLF-D6SC01470D-s001

SC-OLF-D6SC01470D-s002

## Data Availability

CCDC 2514285, 2514457, and 2516780–2516785 contain the supplementary crystallographic data for this paper.^44*a*–*h*^ The data supporting this article have been included as part of the supplementary information (SI). Supplementary information is available. See DOI: https://doi.org/10.1039/d6sc01470d.
