# Supplementary material for: Alkali metal cation effects for rapid C–H activation by iron(0) complexes
Source: Chem Sci. 2026 Mar 31;17(20):10061–6. doi: 10.1039/d6sc01470d (PMC13055520; doi:10.1039/d6sc01470d)
Supplement: SC-017-D6SC01470D-s001 [file SC-017-D6SC01470D-s001.pdf]

Supplementary Information (SI) for Chemical Science.
This journal is © The Royal Society of Chemistry 2026

Electronic Supporting Information

Alkali Metal Cation Effects for Rapid C–H Activation by Iron(0) Complexes

Nereida Hidalgo,[1] Kendal W. Southwell,[1] Ryan S. Donnelly,[1] Brandon Q. Mercado,[1] Sebastian M. Krajewski,[1] Xiaoping Wang,*[,2] and Patrick L. Holland*[,1]

[1] Department of Chemistry, Yale University, New Haven, CT 06520

[2] Neutron Scattering Division, Oak Ridge National Laboratory, Oak Ridge, TN 37831

Table of Contents

General	S-2
Synthesis	S-3
NMR spectra	S-4
UV-vis spectra	S-19
Crystal structures	S-21
Alternative fits of Mössbauer spectra	S-39
References	S-40

General considerations. All reactions were performed under an argon atmosphere in an M. Braun glovebox maintained below 1 ppm of O_2 and H_2O. Glassware was dried at 160 °C. Solvents were dried by passage through Q5 columns from Glass Contour Co., with the exception of THF, which was distilled under Ar from a potassium benzophenone ketyl solution. Toluene-d_8 and C_6D_6 were dried over sodium benzophenone and distilled before use. $L^{Me,iPr}FeC_6H_6$ was prepared as reported.[1] 18-crown-6 was recrystallized from ethyl ether and dried under vacuum. KC_8 and RbC_8 were prepared by heating stoichiometric amounts of rubidium and graphite at 130 °C under an argon atmosphere. CsC_8 was prepared by mixing stoichiometric amounts of cesium and graphite at ambient temperature under argon atmosphere. *Warning: Alkali metals and their graphite intercalates, e.g. KC_8, RbC_8 and CsC_8, are powerful reductants that ignite on contact with air and moisture. Therefore, extreme care must be taken when synthesizing and handling these alkali graphite reductants.*

Instrumentation. NMR spectra were acquired on an Agilent 400 MHz spectrometer. ^1H NMR chemical shifts were referenced to residual ^1H signals from the deuterated solvent with which the sample was prepared. Spectroscopic yields were determined using a capillary containing a solution of nickelocene internal standard. UV-vis spectra were collected on either Cary 60 or Cary 5000 spectrophotometers using Schlenk-adapted quartz cuvettes with a 1 mm optical path length. IR spectra of solids or thin films were obtained using a Bruker Alpha spectrometer containing a diamond ATR unit with 2 cm^{-1} resolution. Mössbauer data were recorded on a SEECo spectrometer with alternating constant acceleration; isomer shifts are relative to iron metal at 298 K. The sample temperature was maintained at 80 K in a Janis Research Company Inc. cryostat. The zero-field spectra were simulated as Lorentzian doublets using WMoss (SeeCo).

Synthesis of complexes 1 (K), 2 (Rb), and 3 (Cs):

A solution of $L^{Me,iPr}FeC_6H_6$ (100 mg, 0.181 mmol, 1 equiv) in 3 mL of toluene was added to a suspension of 1 equiv of the corresponding AMC_8 in 2 mL of toluene. After 15 min, the crude was filtered through a plug of Celite and concentrated to *ca.* 2 mL. This solution was cooled to –40 ºC, and crystals formed over 16 h. They were collected via filtration and dried under vacuum to give a purple solid. Typical yields were 30-50%. When these were dissolved in C_6D_6 for NMR spectroscopy, some impurities began to form almost immediately. Considering the very low stability, it is unlikely that any samples have high purity, which should be taken into consideration when interpreting the spectroscopic data.

Complex **1** (**K**): ^{1}H NMR (400 MHz, 298 K, C_6D_6): δ 79 (4H), 17 (12H), -46 (4H), -79 (12H) ppm. The rest of the peaks could overlapped or absent, and we were unable to assign them, though some small peaks are indicative of impurities (Figure S4). Zero-field Mössbauer (solid, 80 K): δ = 0.48 mm s^{-1}, $|\Delta E_Q|$ = 2.65 mm s^{-1}. IR (ATR, solid): 2955 (m), 2925 (m), 2865 (m), 1533 (s), 1402 (s), 1314 (s), 1249 (m), 1171(m), 1098 (m), 1020 (m), 931 (w), 794 (s), 762 (s), 713 (s), 463 (w) cm^{-1}.

Complex **2** (**Rb**): ^{1}H NMR (400 MHz, 298 K, C_6D_6): δ 74 (4H), 17 (12H), -47 (4H), -81 (12H) ppm. The rest of the peaks could were overlapped or absent, and we were unable to assign them, though some small peaks are indicative of impurities (Figure S5). Zero-field Mössbauer (solid, 80 K): δ = 0.49 mm s^{-1}, $|\Delta E_Q|$ = 2.69 mm s^{-1}. IR (ATR, solid): 3048 (w), 2958 (m), 1513 (w), 1385 (s), 1318(s), 1169 (m), 1100 (m), 932 (m), 848 (m), 792 (m), 758 (m), 709 (m), 621 (w), 595 (w), 524 (w), 438 (w) cm^{-1}.

Complex **3** (**Cs**): ^{1}H NMR (400 MHz, 298 K, C_6D_6): δ 72 (4H), 22 (12H), -48 (4H), -84 (12H) ppm. The rest of the peaks could were overlapped or absent, and we were unable to assign them, though some small peaks are indicative of impurities (Figure S6). Zero-field Mössbauer (solid, 80 K): δ_1 = 0.49 mm s^{-1}, $|\Delta E_{Q1}|$ = 2.66 mm s^{-1}. Unknown byproduct (10% of the sample): δ_2 = -0.23 mm s^{-1}, $|\Delta E_{Q2}|$ = 1.18 mm s^{-1}. IR (ATR, solid): 3048 (w), 2959 (m), 2862 (m), 1513 (s), 1437 (s), 1395, 1317 (s), 1254 (m), 1168 (s), 1099 (s), 1052 (s), 1023 (s), 930 (s), 796, 763, 711, 527 (w), 443 (s) cm^{-1}.

NMR Spectra

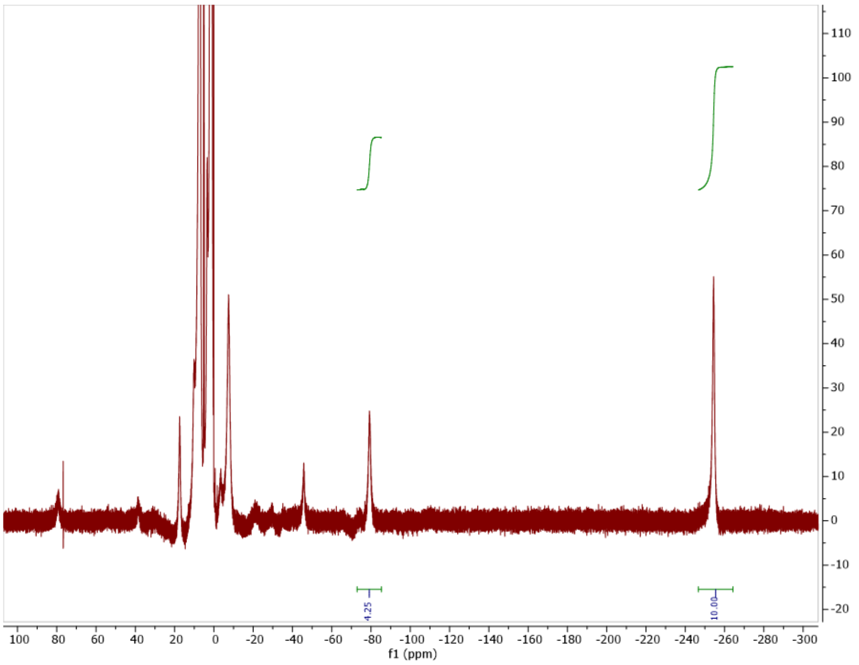

Figure S1. ^{1}H NMR spectrum of the crude **1 (K)** sample integrated with respect to a 12.9 mM nickelocene capillary in toluene-d_8 (δ -252 ppm, 10H). Spectroscopic yield of **1** (**K**) is 36%.

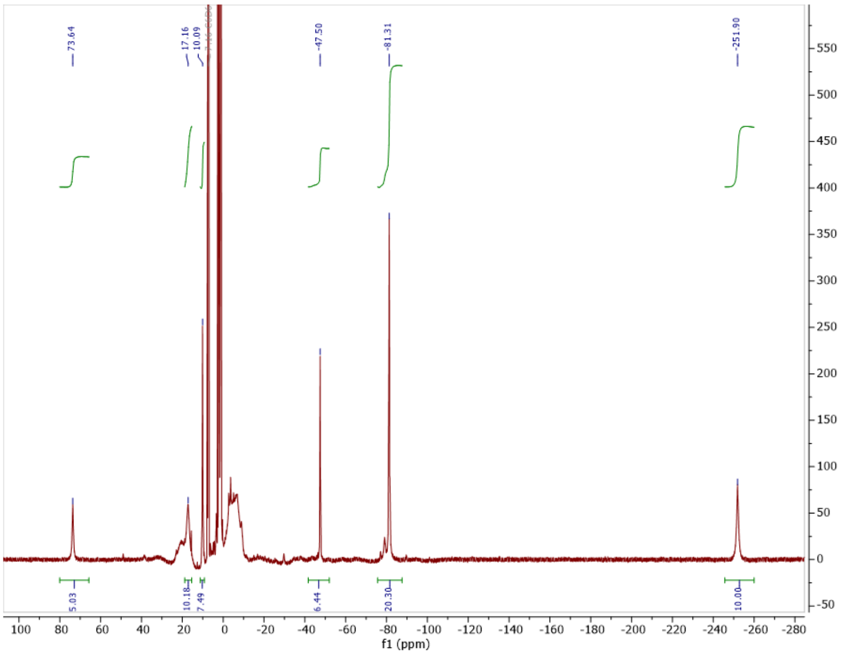

Figure S2. ^{1}H NMR spectrum of the crude **2 (Rb)** sample integrated with respect to a 4.5 mM nickelocene capillary in toluene-d_8 (δ -252 ppm, 10H). Spectroscopic yield of **2** (**Rb**) is 47%.

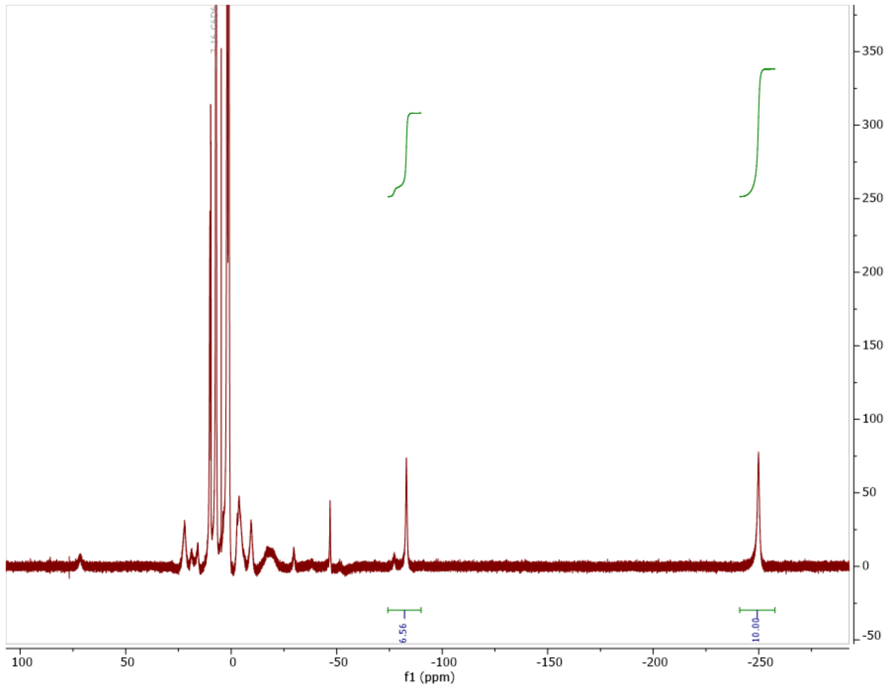

Figure S3. ^{1}H NMR spectrum of the crude **3 (Cs)** sample integrated with respect to a 12.9 mM nickelocene capillary in toluene-d_8 (δ -252 ppm, 10H). Spectroscopic yield of **3** (**Cs**) is 47%.

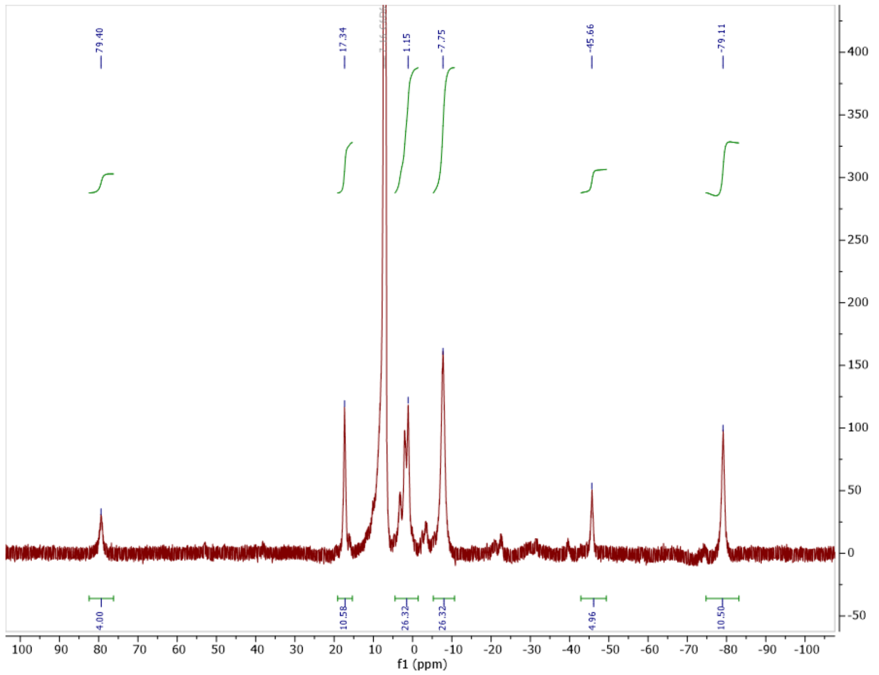

Figure S4. ^{1}H NMR spectrum of **1 (K)** in C_6D_6.

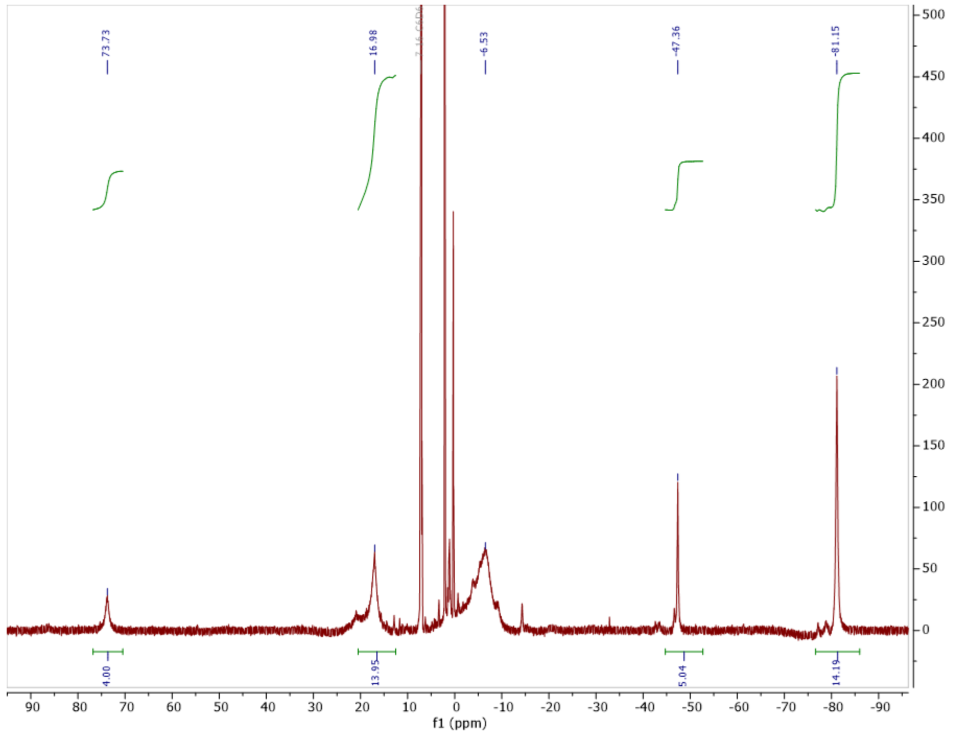

Figure S5. ^{1}H NMR spectrum of **2 (Rb)** in C_6D_6.

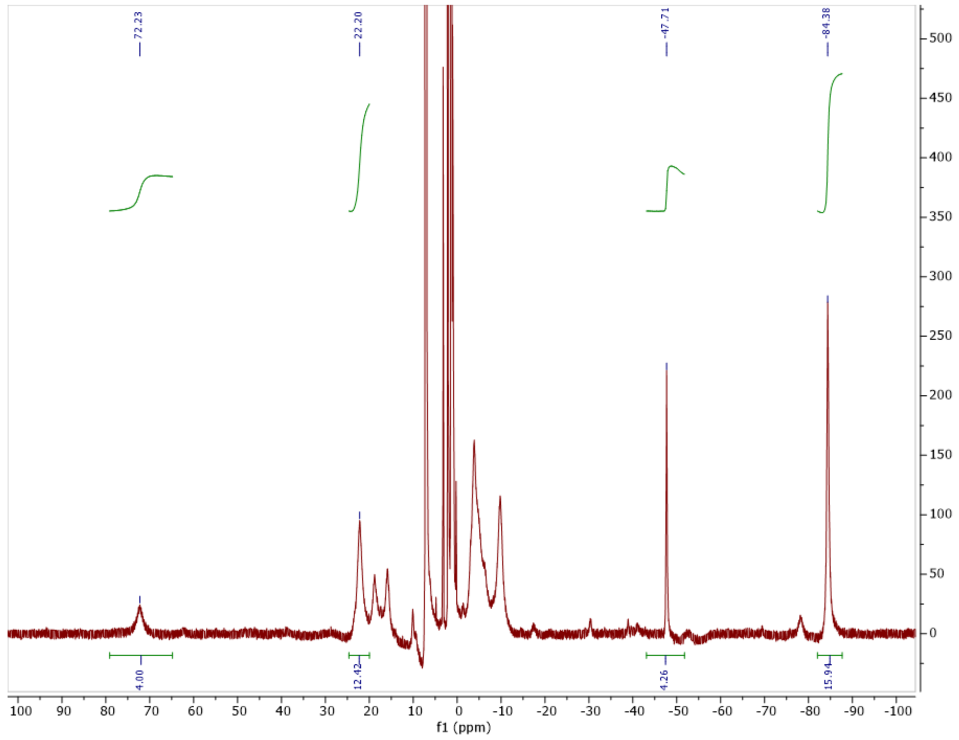

Figure S6. ^{1}H NMR spectrum of **3 (Cs)** in C_6D_6.

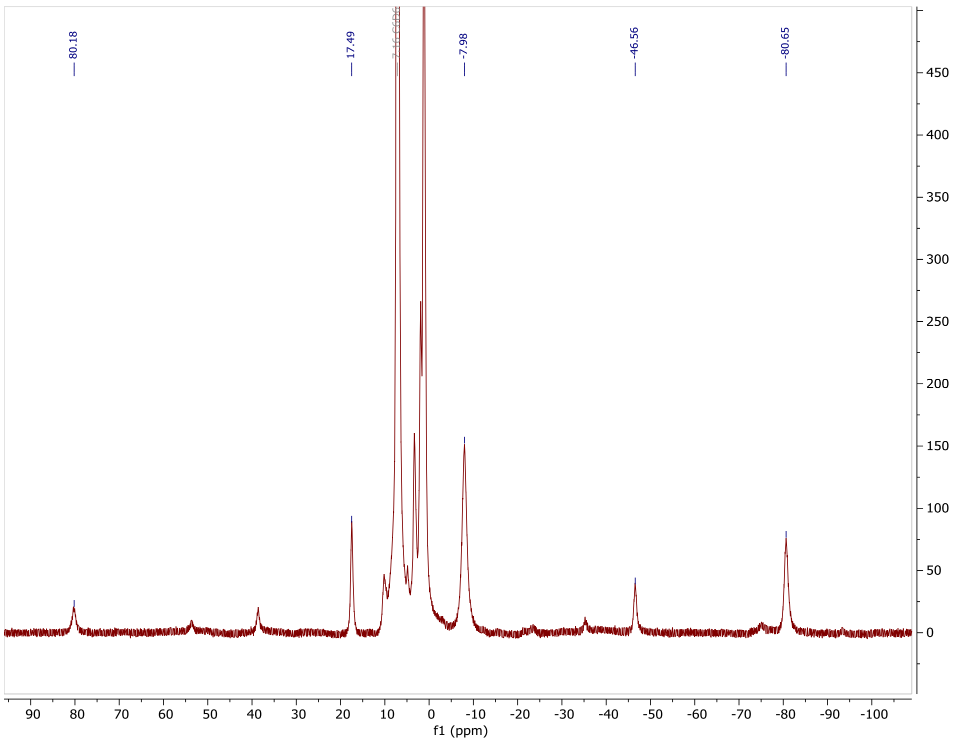

Figure S7. ^{1}H NMR spectrum of **1 (K)** in C_6D_6 synthesized using an excess of KC_8**.**

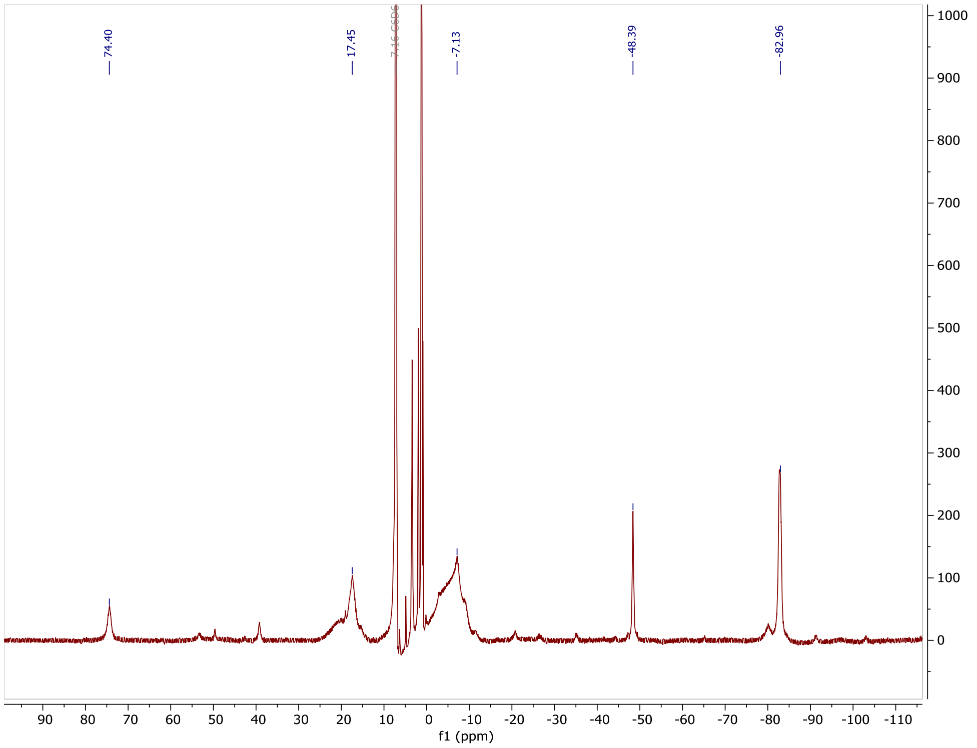

Figure S8. ^{1}H NMR spectrum of **2 (Rb)** in C_6D_6 synthesized using an excess of RbC_8**.**

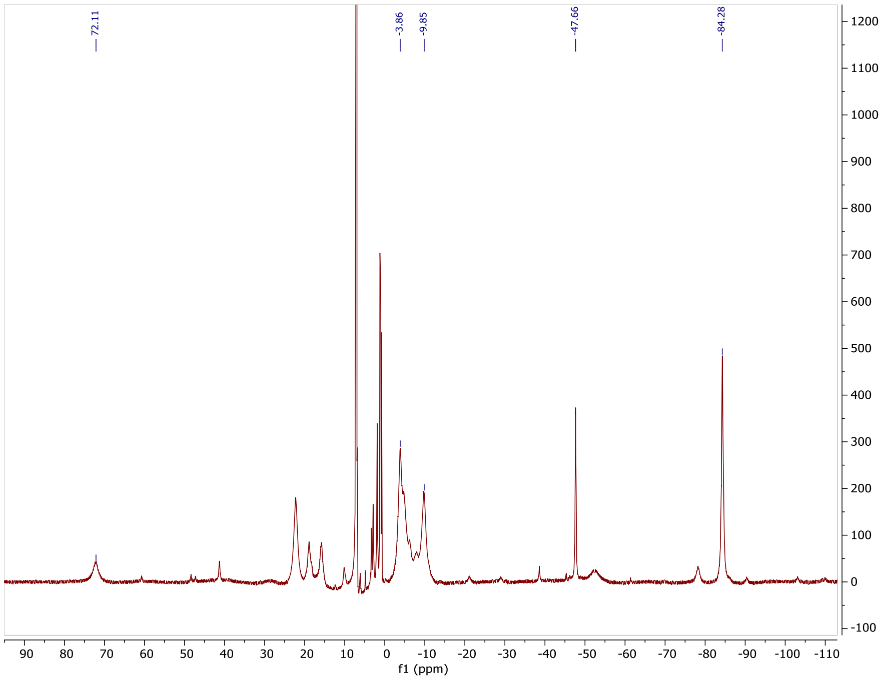

Figure S9. ^{1}H NMR spectrum of **3 (Cs)** in C_6D_6 synthesized using an excess of CsC_8**.**

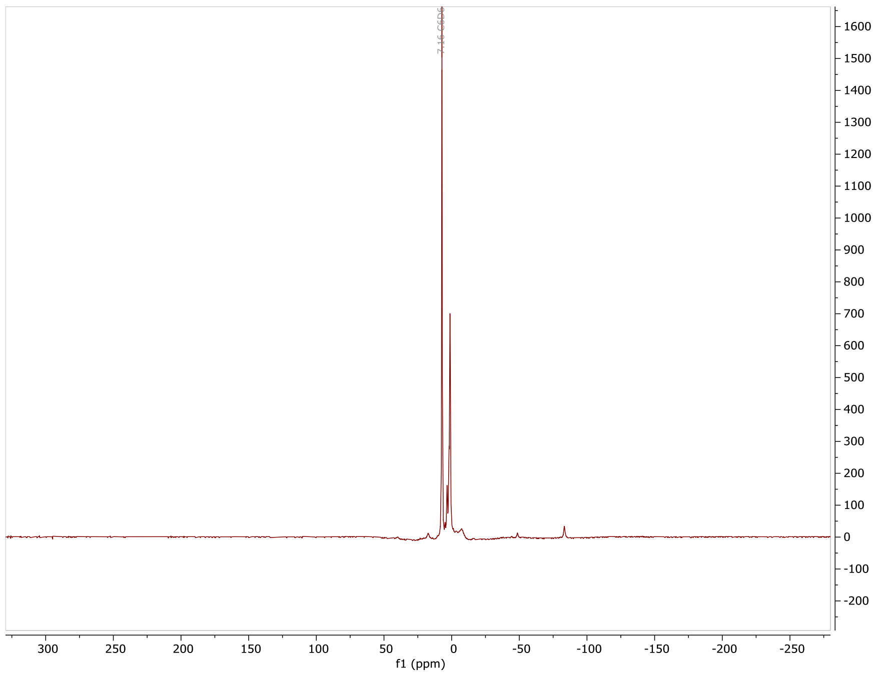

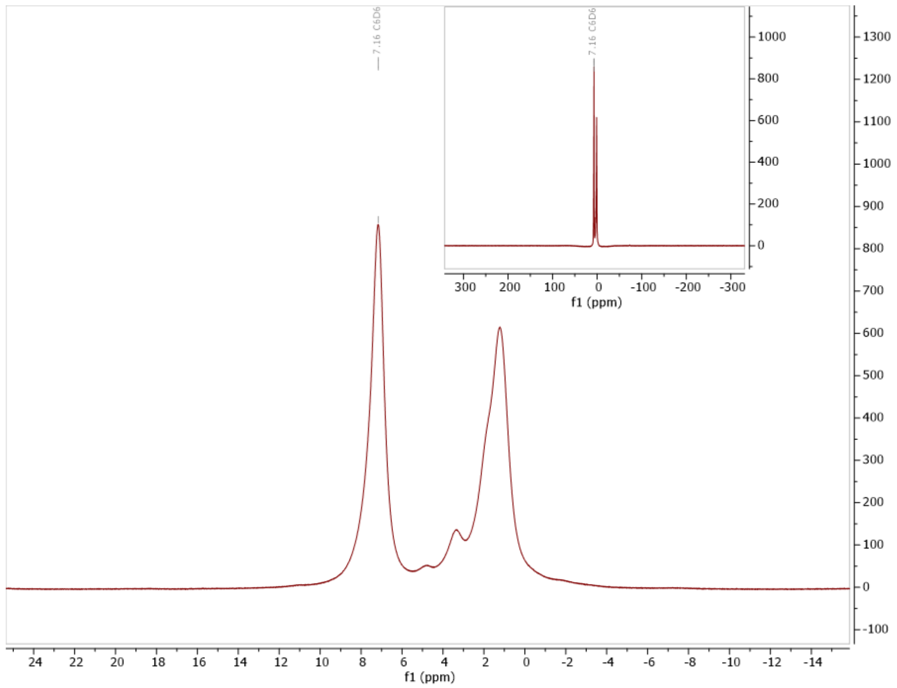

Figure S10. ^{1}H NMR spectra of **1 (K)** in C_6D_6 after 8 hours at room temperature.

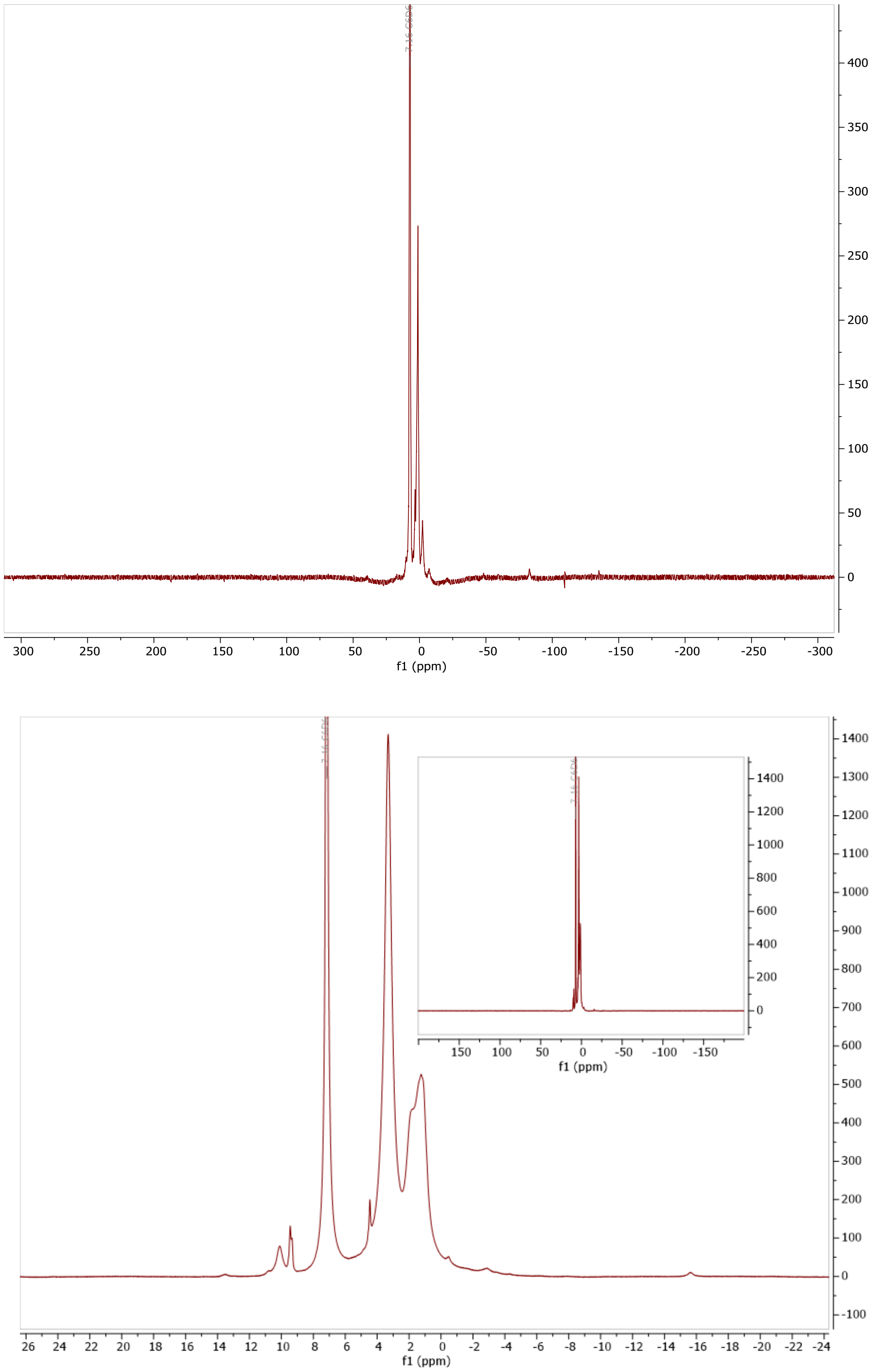

Figure S11. ^{1}H NMR spectra of **2 (Rb)** in C_6D_6 after 8 hours at room temperature.

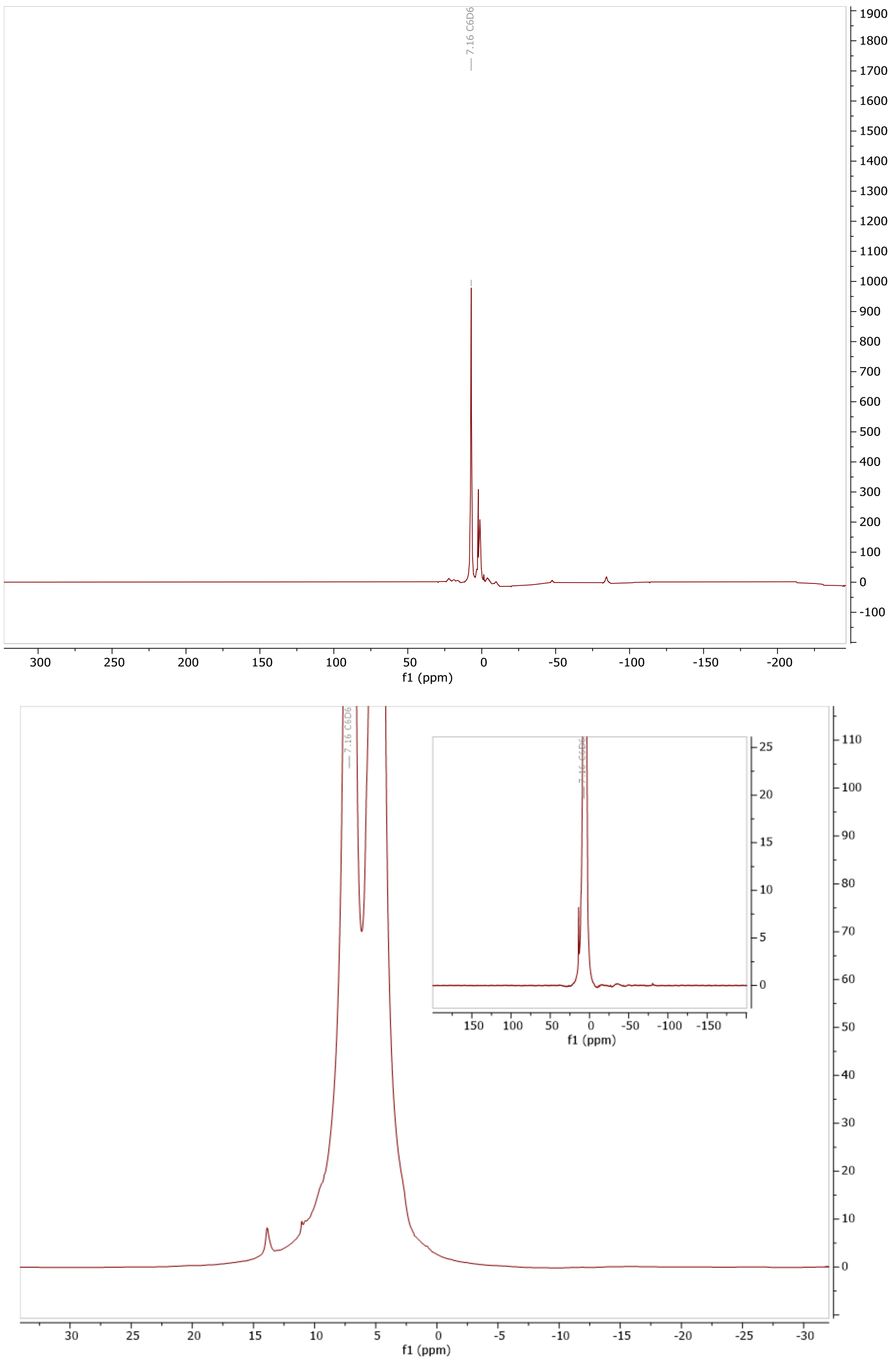

Figure S12. ^{1}H NMR spectra of **3 (Cs)** in C_6D_6 after 8 hours at room temperature.

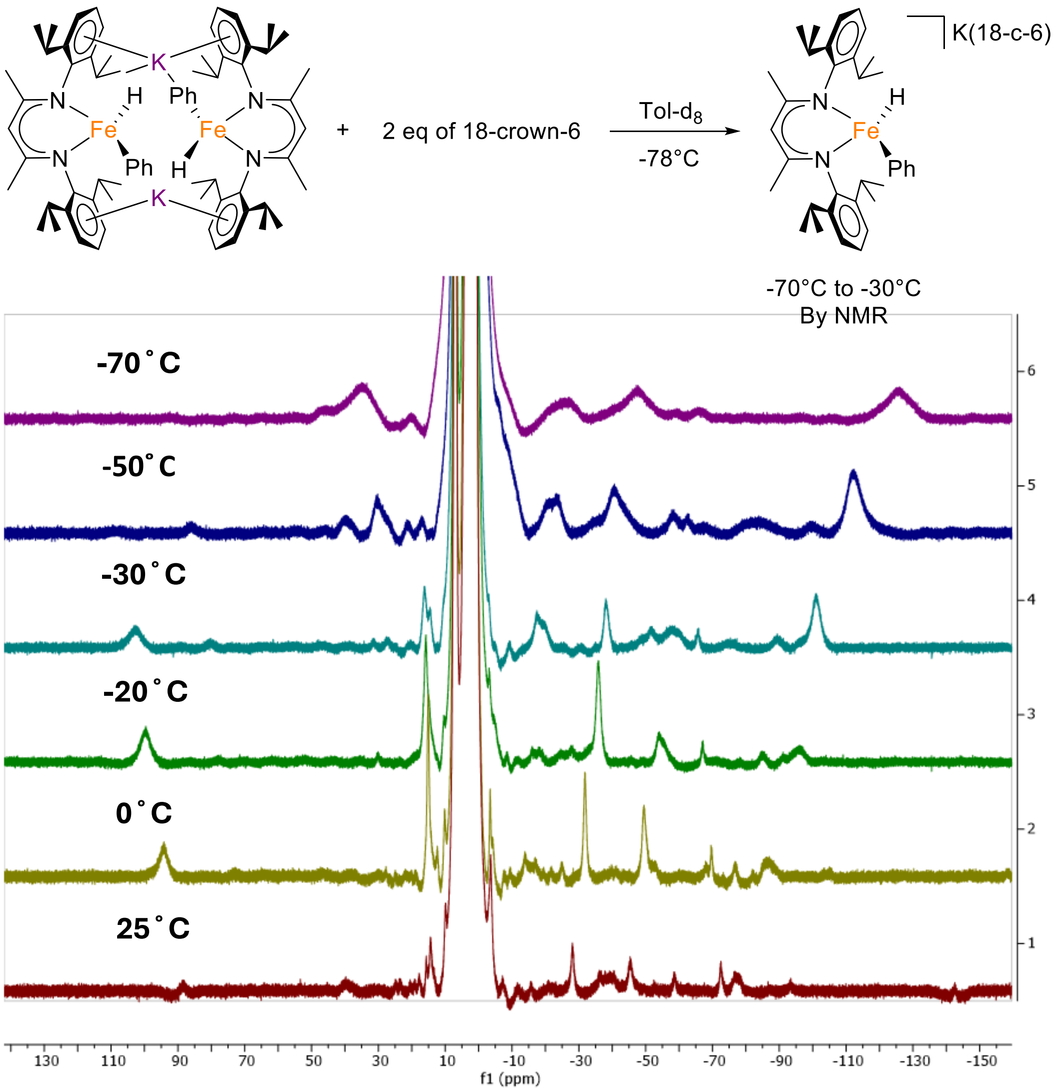

Figure S13. ^{1}H NMR spectra of **1 (K)** with 2 equiv of 18-crown-6 at various temperatures in toluene-d_8, showing formation of LFe(H)(Ph)K(18c6) at low temperatures (-70˚C to -30˚C) with degradation onset around -20˚C.

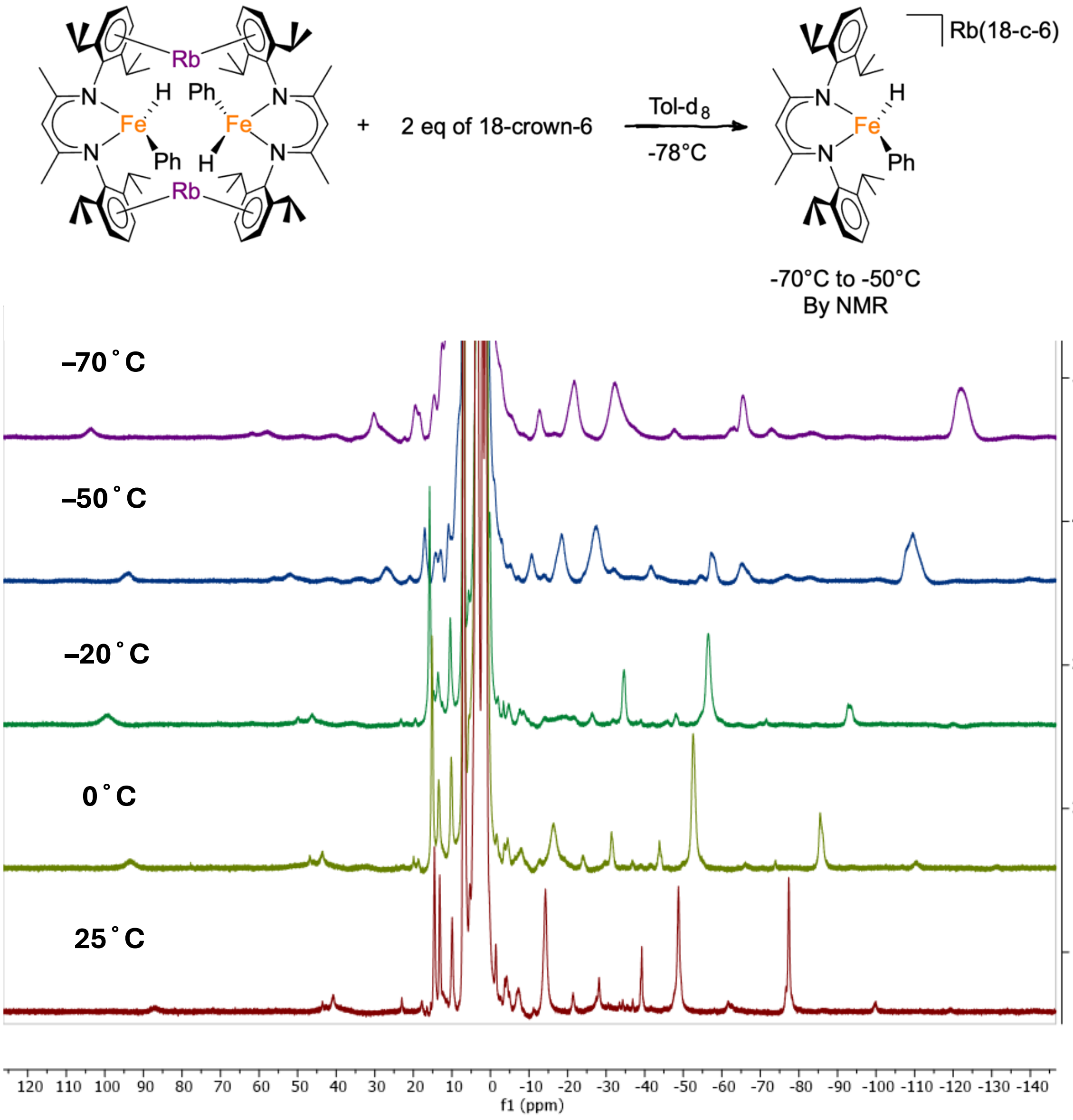

Figure S14. ^{1}H NMR spectra of **2 (Rb)** with 2 equiv of 18-crown-6 at various temperatures in toluene-d_8, showing formation of LFe(H)(Ph)Rb(18c6) at low temperatures (-70˚C to -30˚C) with degradation onset around -20˚C.

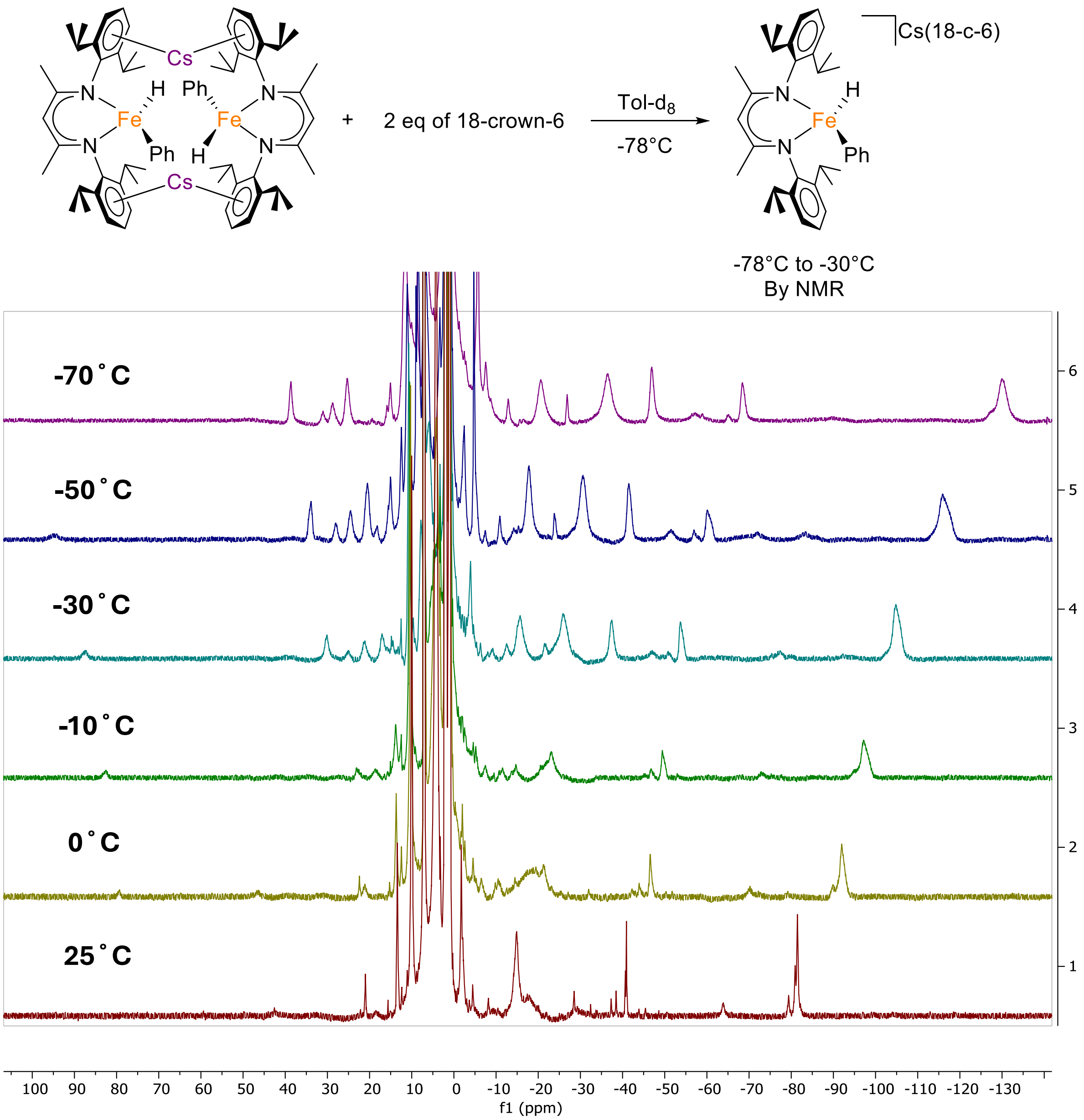

Figure S15. ^{1}H NMR spectra of **3 (Cs)** with 2 equiv of 18-crown-6 at various temperatures in toluene-d_8, showing formation of LFe(H)(Ph)Cs(18c6) at low temperatures (-70˚C to -30˚C) with degradation onset around -10˚C.

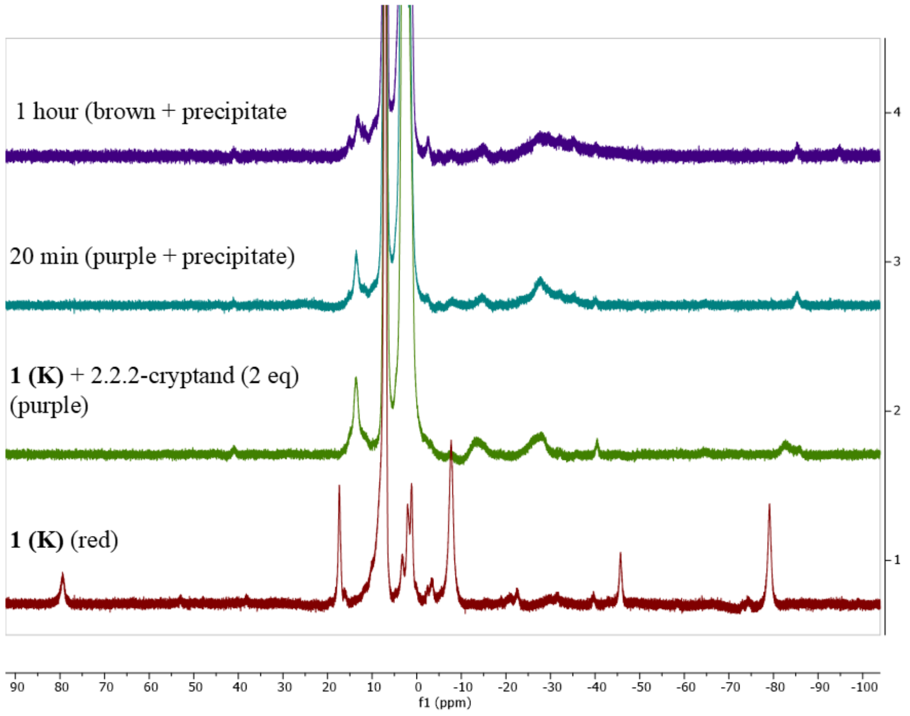

Figure S16. ^{1}H NMR spectrum of **1 (K)** with 2 equiv of 2.2.2-cryptand in C_6D_6 at 25˚C.

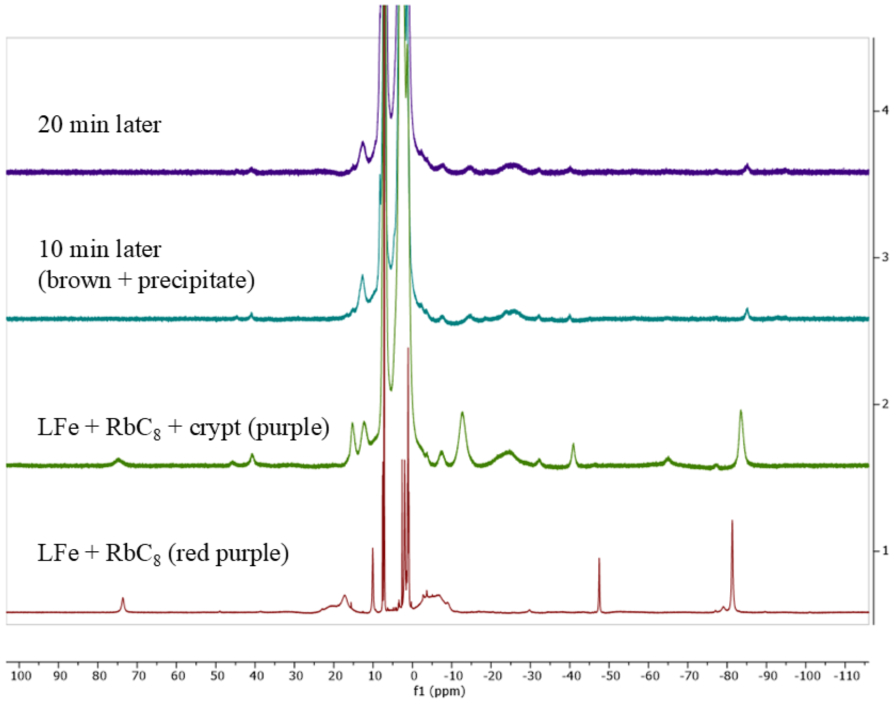

Figure S17. ^{1}H NMR spectrum of **2 (Rb)** with 2 equiv of 2.2.2-cryptand in C_6D_6 at 25˚C.

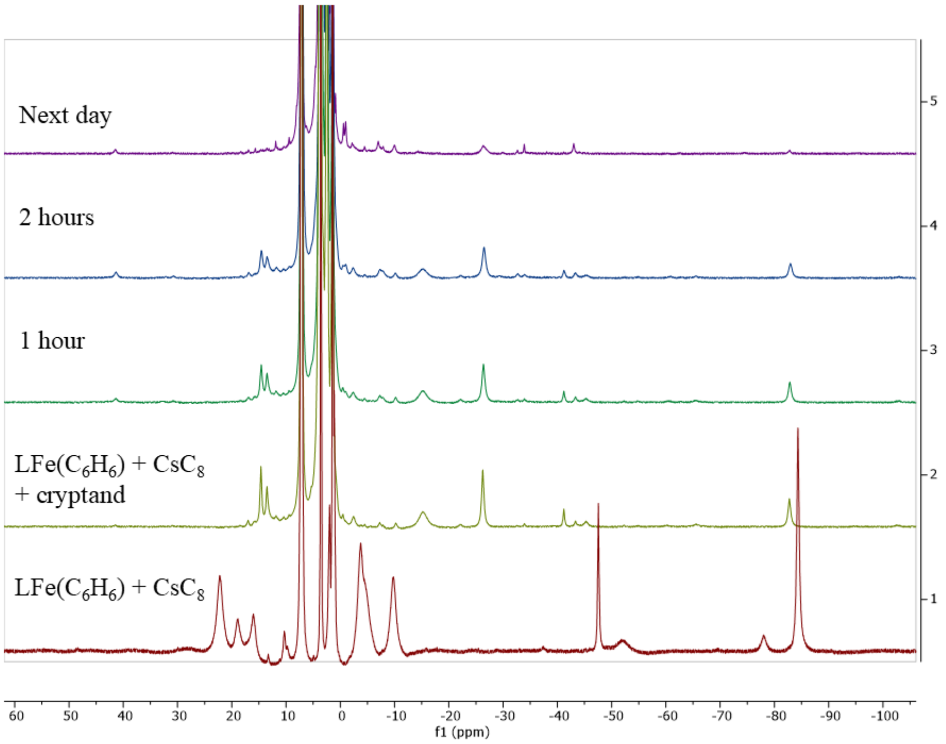

Figure S18. In situ ^{1}H NMR spectrum of **3 (Cs)** with 2 equiv of 2.2.2-cryptand in C_6D_6 at 25˚C.

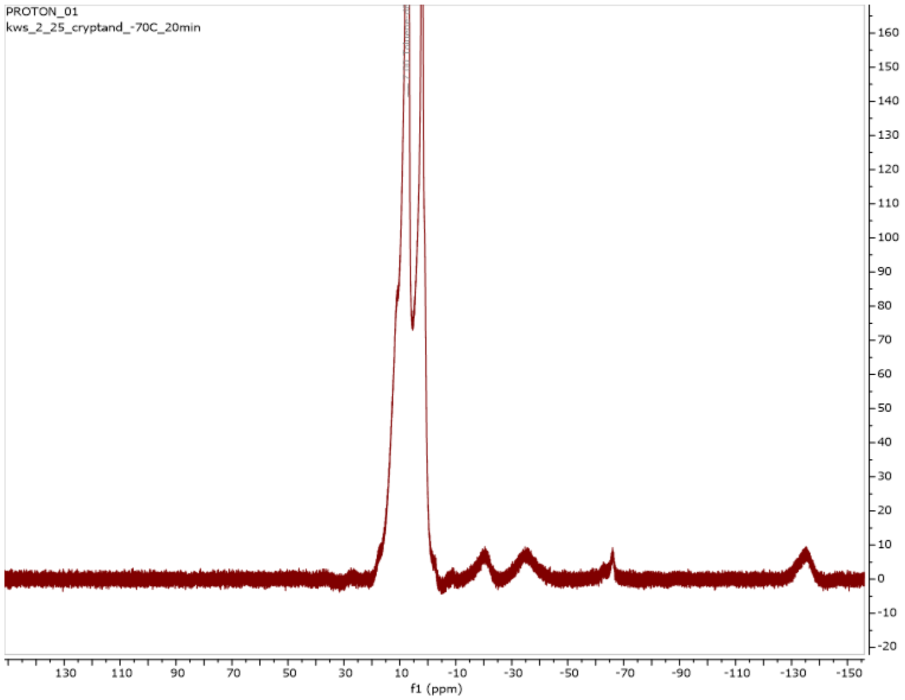

Figure S19. ^{1}H NMR spectra of **2 (Rb)** with 2 equiv of 2.2.2-cryptand at –70˚C in toluene-d_8.

IR spectra

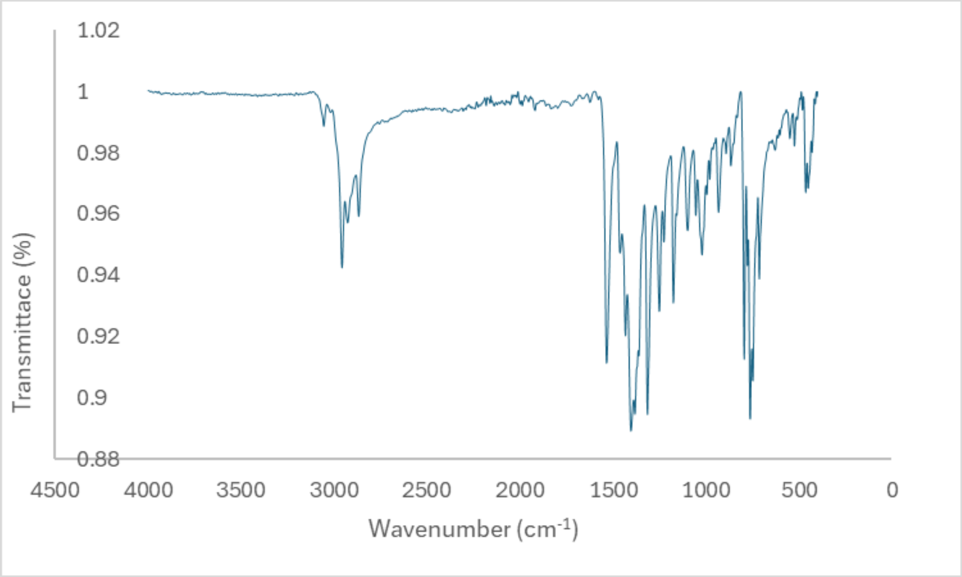

Figure S20. IR spectrum (ATR) of **1** (**K**).

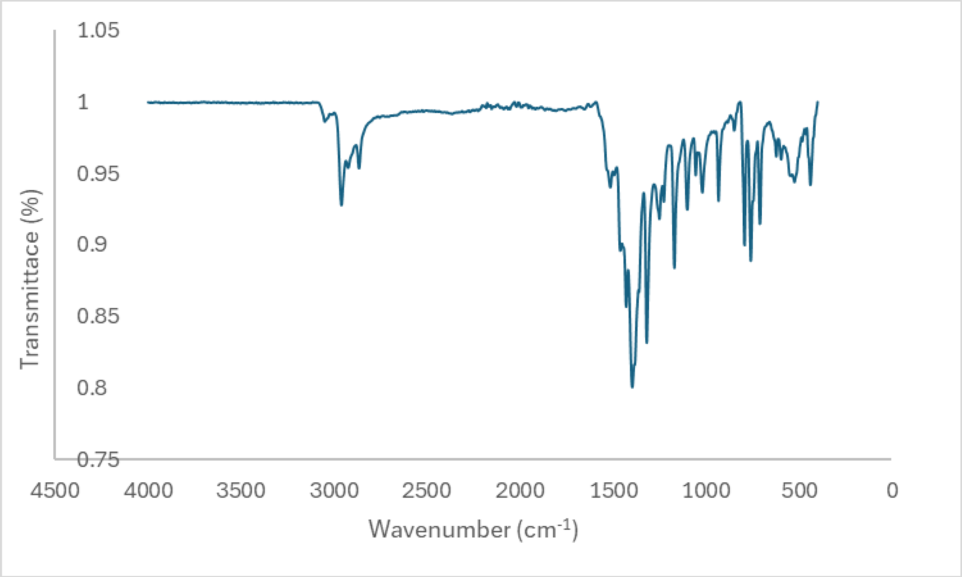

Figure S21. IR spectrum (ATR) of **2** (**Rb**).

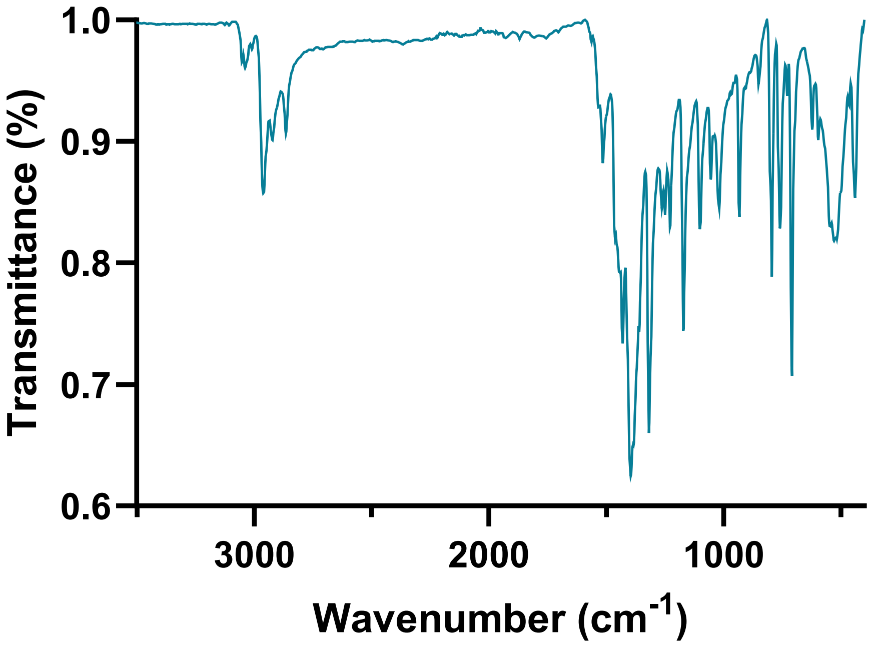

Figure S22. IR spectrum (ATR) of **3** (**Cs**).

UV-Vis spectra

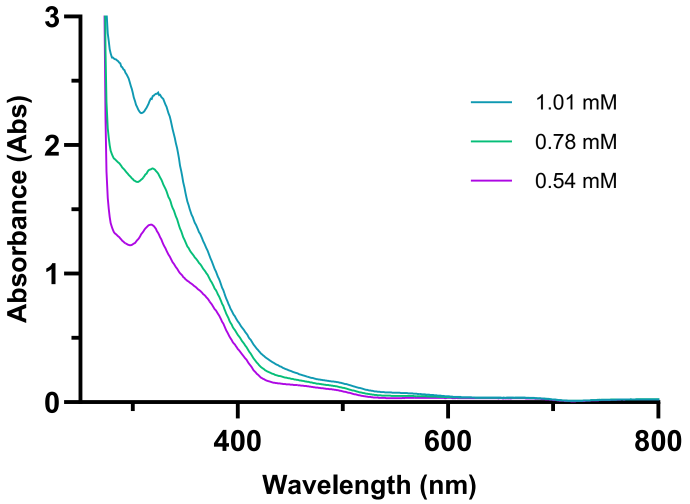

Figure S23. Electronic absorption spectra of **1 (K)** in benzene in cuvettes with 0.1 cm path length with concentrations ranging from 0.78 mM to 1.01 mM.

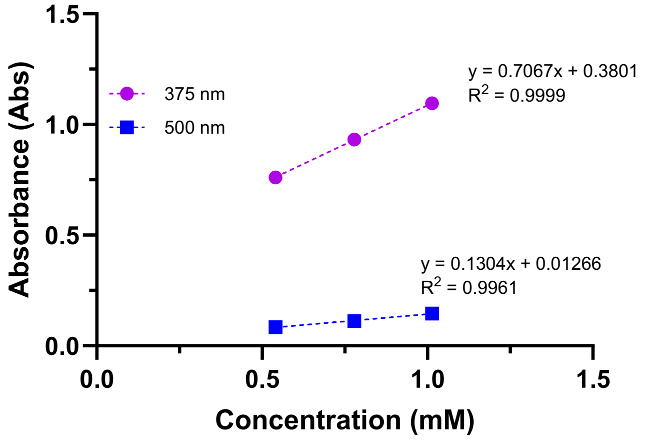

Figure S24. Beer-Lambert Law plot of selected UV-Vis spectral features of **1 (K)** in benzene solutions using cuvettes with 0.1 cm path length. There are likely impurities and the intercepts are away from zero, so the values should be viewed with caution.

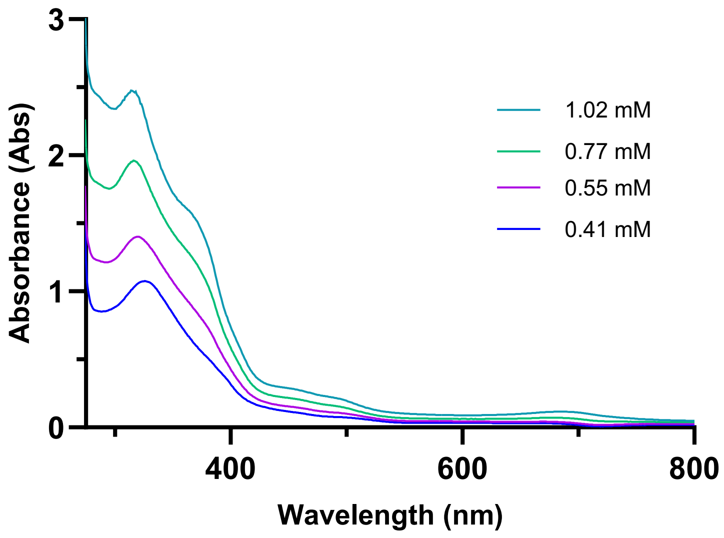

Figure S25. Electronic absorption spectra of **2 (Rb)** in benzene in cuvettes with 0.1 cm path length with concentrations ranging from 0.41 mM to 1.02 mM.

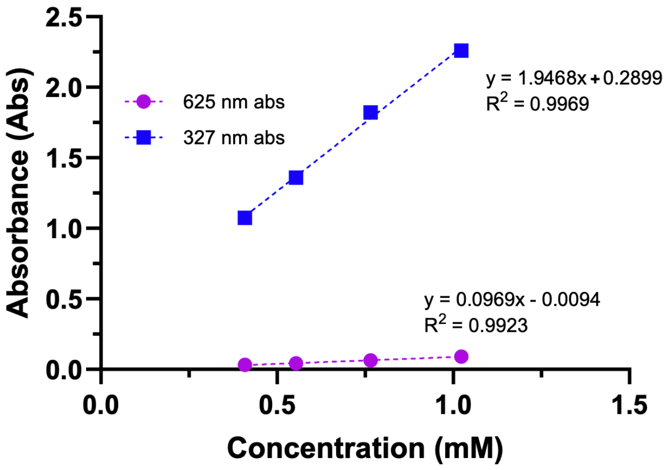

Figure S26. Beer-Lambert Law plot of selected UV-Vis spectral features of **2 (Rb)** in benzene solutions using cuvettes with 0.1 cm path length. There are likely impurities and the intercepts are away from zero, so the values should be viewed with caution. ε = 19,000 $M^{-1}cm^{-1}$ at 327 nm and 1000 $M^{-1}cm^{-1}$ at 625 nm.

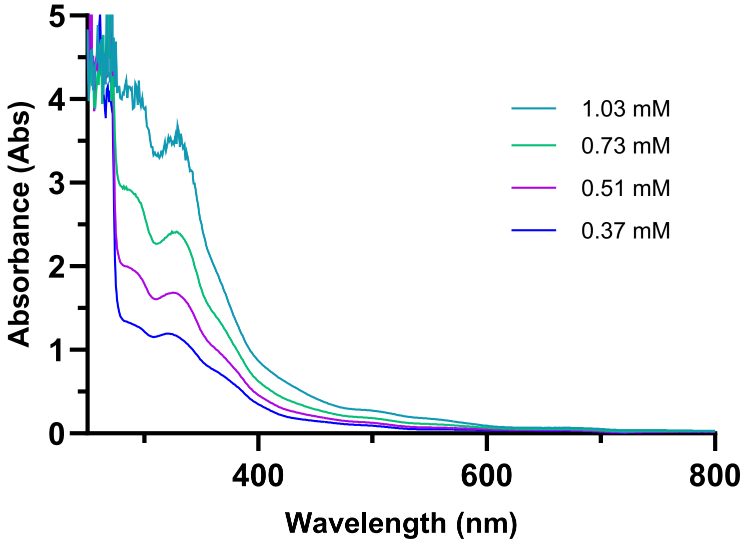

Figure S27. Electronic absorption spectra of **3 (Cs)** in benzene in cuvettes with 0.1 cm path length with concentrations ranging from 0.37 mM to 1.03 mM.

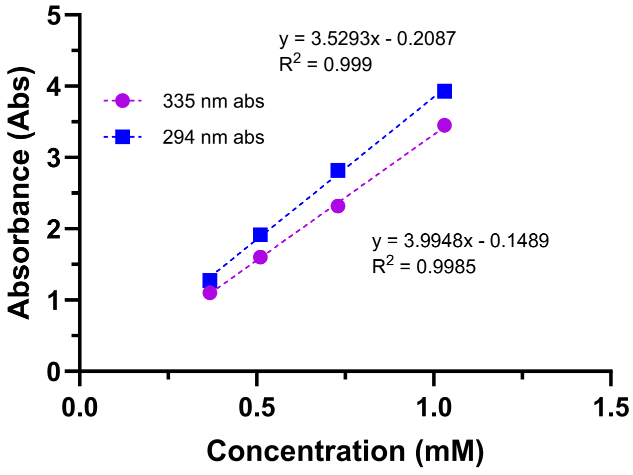

Figure S28. Beer-Lambert Law plot of selected UV-Vis spectral features of **3 (Cs)** in benzene solutions using cuvettes with 0.1 cm path length. There are likely impurities and the intercepts are away from zero, so the values should be viewed with caution. ε = 35,000 $M^{-1}cm^{-1}$ at 294 nm and 40,000 $M^{-1}cm^{-1}$ at 335 nm.

Crystallography

Complex **1 (K)**

Low-temperature diffraction data (ω-scans) were collected on a Rigaku Synergy-S diffractometer coupled to a HyPix-Arc 100 detector with Cu Kα (λ = 1.54178 Å) for the structure of syn-24078. The diffraction images were processed and scaled using Rigaku Oxford Diffraction software (CrysAlisPro; Rigaku OD: The Woodlands, TX, 2015). The structure was solved with SHELXT and was refined against F^2 on all data by full-matrix least squares with SHELXL (Sheldrick, G. M. Acta Cryst. 2008, A64, 112–122). All non-hydrogen atoms were refined anisotropically. Hydrogen atoms were included in the model at geometrically calculated positions and refined using a riding model, except for the hydride which was refined freely. The isotropic displacement parameters of all hydrogen atoms were fixed to 1.2 times the U value of the atoms to which they are linked (1.5 times for methyl groups). CCDC number 2516782 contains the detailed crystallographic data for this structure.

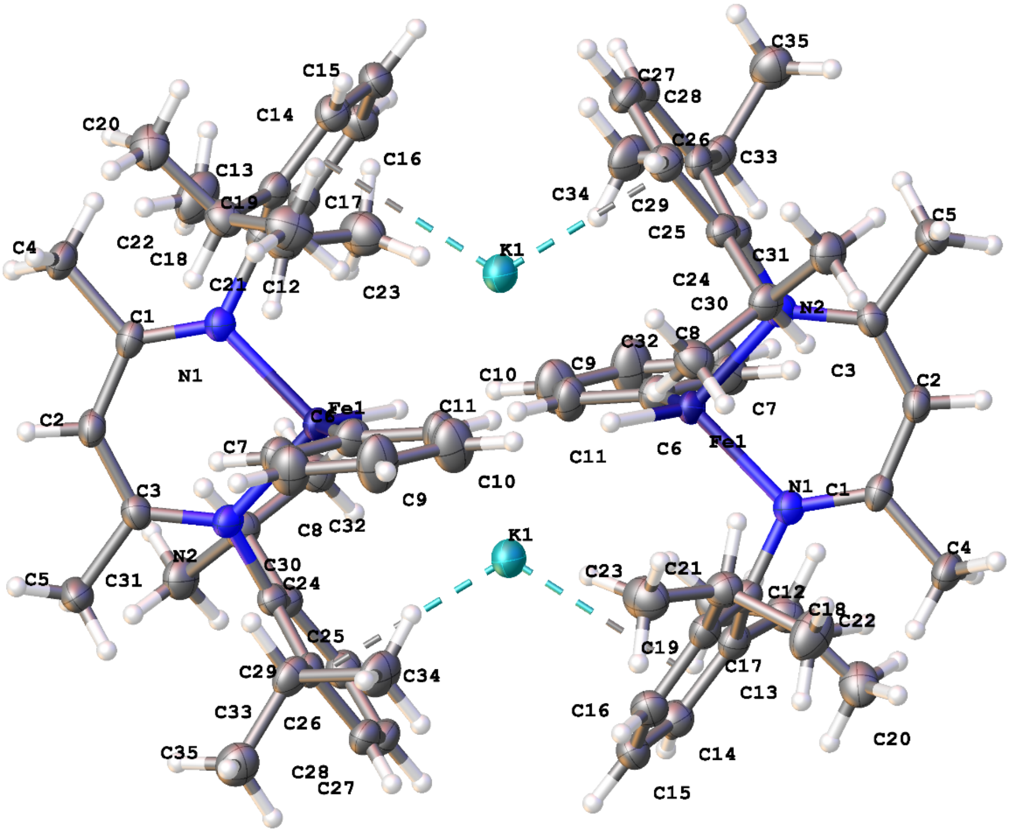

Figure S29. The complete numbering scheme of **1 (K)** with 50% thermal ellipsoid probability levels. The hydrogen atoms are shown as circles rather than thermal ellipsoids.

Table S1. Crystal data and structure refinement for **1 (K)**.

Identification code	syn-24078	
Empirical formula	C70 H94 Fe2 K2 N4	
Formula weight	1181.39	
Temperature	108.7(3) K	
Wavelength	1.54184 Å	
Crystal system	Triclinic	
Space group	P-1	
Unit cell dimensions	a = 12.3196(3) Å	α= 66.048(3)°.
	b = 12.4076(3) Å	β= 65.925(3)°.
	c = 13.5563(4) Å	γ = 62.061(3)°.
Volume	1611.03(10) $Å^3$	
Z	1	
Density (calculated)	1.218 g/cm^3	
Absorption coefficient	5.080 mm^{-1}	
F(000)	632	
Crystal size	0.16 x 0.1 x 0.02 mm^3	
Crystal color and habit	red plate	
Diffractometer	XtaLAB Synergy, Dualflex, HyPix-Arc 100	
Theta range for data collection	3.705 to 79.000°.	
Index ranges	-15<=h<=15, -15<=k<=15, -17<=l<=16	
Reflections collected	45957	
Independent reflections	6833 [R(int) = 0.0670]	
Observed reflections (I > 2sigma(I))	6612	
Completeness to theta = 67.684°	99.8 %	
Absorption correction	Semi-empirical from equivalents	
Max. and min. transmission	1.00000 and 0.45709	
Solution method	SHELXT 2018/2 (Sheldrick, 2018)	
Refinement method	SHELXL 2019/3 (Sheldrick, 2015)	
Data / restraints / parameters	6833 / 0 / 367	
Goodness-of-fit on F^2	1.119	
Final R indices [I>2sigma(I)]	R1 = 0.0879, wR2 = 0.2368	
R indices (all data)	R1 = 0.0893, wR2 = 0.2375	
Extinction coefficient	0.0025(4)	
Largest diff. peak and hole	0.974 and -0.595 e.$Å^{-3}$	

Complex **2 (Rb)**

Low-temperature diffraction data (ω-scans) were collected on a Rigaku MicroMax-007HF diffractometer coupled to a Dectris Pilatus3R detector with Mo Kα (λ = 0.71073 Å) for the structure of 007c-24010. The diffraction images were processed and scaled using Rigaku Oxford Diffraction software (CrysAlisPro; Rigaku OD: The Woodlands, TX, 2015). The structure was solved with SHELXT and was refined against F2 on all data by full-matrix least squares with SHELXL (Sheldrick, G. M. Acta Cryst. 2008, A64, 112–122). Hydrogen atoms were included in the model at geometrically calculated positions and refined using a riding model, except for the hydride which was refined freely. The isotropic displacement parameters of all hydrogen atoms were fixed to 1.2 times the U value of the atoms to which they are linked (1.5 times for methyl groups). CCDC number 2516781 contains the detailed crystallographic data for this structure.

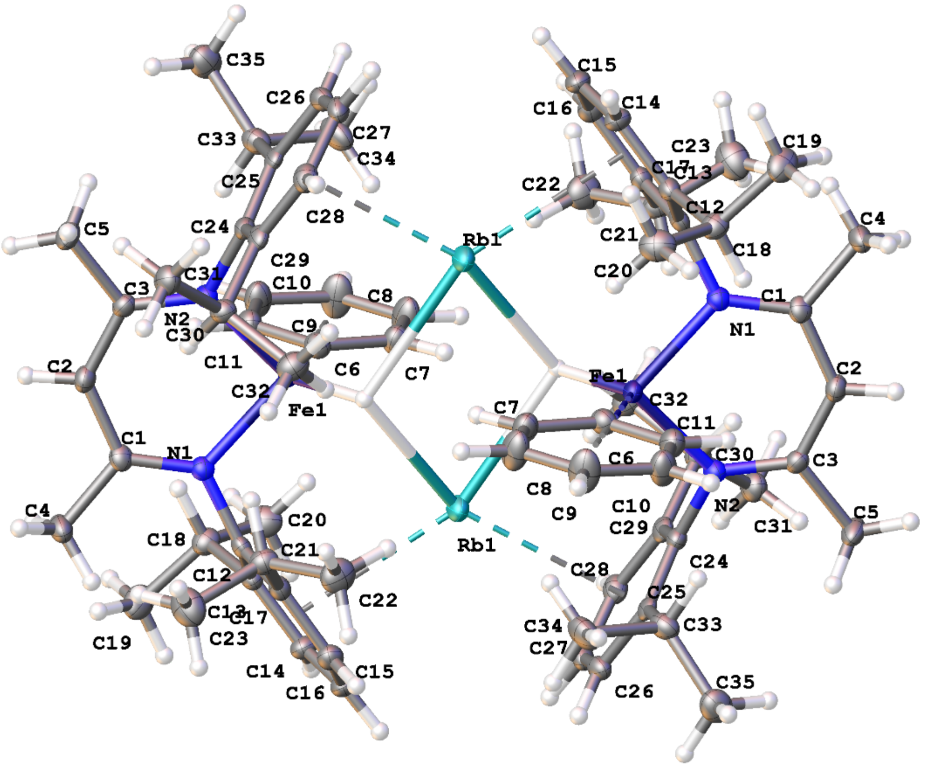

Figure S30. The complete numbering scheme of **2 (Rb)** with 50% thermal ellipsoid probability levels. The hydrogen atoms are shown as circles rather than thermal ellipsoids.

Table S2. Crystal data and structure refinement for **2 (Rb)**.

Identification code	007c-24010	
Empirical formula	C70 H94 Fe2 N4 Rb2	
Formula weight	1274.13	
Temperature	99.98(16) K	
Wavelength	0.71073 Å	
Crystal system	Triclinic	
Space group	P-1	
Unit cell dimensions	a = 12.3100(4) Å	α= 66.204(4)°.
	b = 12.4451(4) Å	β= 66.310(4)°.
	c = 13.6698(6) Å	γ = 62.018(4)°.
Volume	1632.61(13) $Å^3$	
Z	1	
Density (calculated)	1.296 g/cm^3	
Absorption coefficient	1.968 mm^{-1}	
F(000)	668	
Crystal size	0.11 x 0.1 x 0.09 mm^3	
Crystal color and habit	red block	
Diffractometer	XtaLAB AFC12 (RCD3): Kappa single	
Theta range for data collection	2.876 to 30.242°.	
Index ranges	-16<=h<=16, -16<=k<=16, -18<=l<=19	
Reflections collected	44303	
Independent reflections	8336 [R(int) = 0.0992]	
Observed reflections (I > 2sigma(I))	6160	
Completeness to theta = 25.242°	99.8 %	
Absorption correction	Semi-empirical from equivalents	
Max. and min. transmission	1.00000 and 0.68445	
Solution method	SHELXT 2018/2 (Sheldrick, 2018)	
Refinement method	SHELXL 2019/3 (Sheldrick, 2015)	
Data / restraints / parameters	8336 / 0 / 367	
Goodness-of-fit on F^2	1.212	
Final R indices [I>2sigma(I)]	R1 = 0.0626, wR2 = 0.1535	
R indices (all data)	R1 = 0.0935, wR2 = 0.1610	
Extinction coefficient	0.0043(10)	
Largest diff. peak and hole	1.123 and -0.803 e.$Å^{-3}$	

Complex **3 (Cs)**

Low-temperature diffraction data (ω-scans) were collected on a Rigaku Synergy-S diffractometer coupled to a HyPix-Arc 100 detector with Cu Kα (λ = 1.54178 Å) for the structure of syn-24026. The diffraction images were processed and scaled using Rigaku Oxford Diffraction software (CrysAlisPro; Rigaku OD: The Woodlands, TX, 2015). The structure was solved with SHELXT and was refined against F^2 on all data by full-matrix least squares with SHELXL (Sheldrick, G. M. Acta Cryst. 2008, A64, 112–122). All non-hydrogen atoms were refined anisotropically. Hydrogen atoms were included in the model at geometrically calculated positions and refined using a riding model, except for the hydride which was refined freely. The isotropic displacement parameters of all hydrogen atoms were fixed to 1.2 times the U value of the atoms to which they are linked (1.5 times for methyl groups). CCDC number 2516783 contains the detailed crystallographic data for this structure.

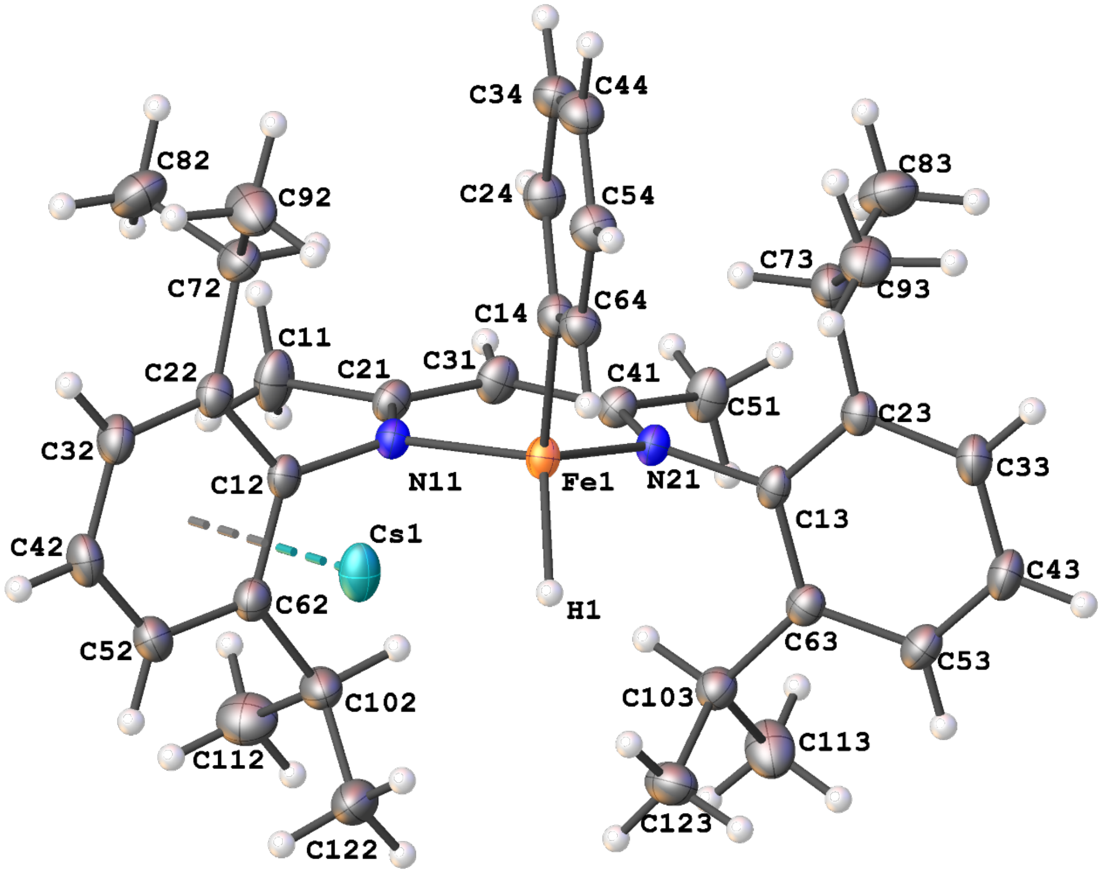

Figure S31. The complete numbering scheme of syn-24026 with 50% thermal ellipsoid probability levels. The asymmetric unit is shown, which is half of the molecule. The hydrogen atoms are shown as circles rather than thermal ellipsoids.

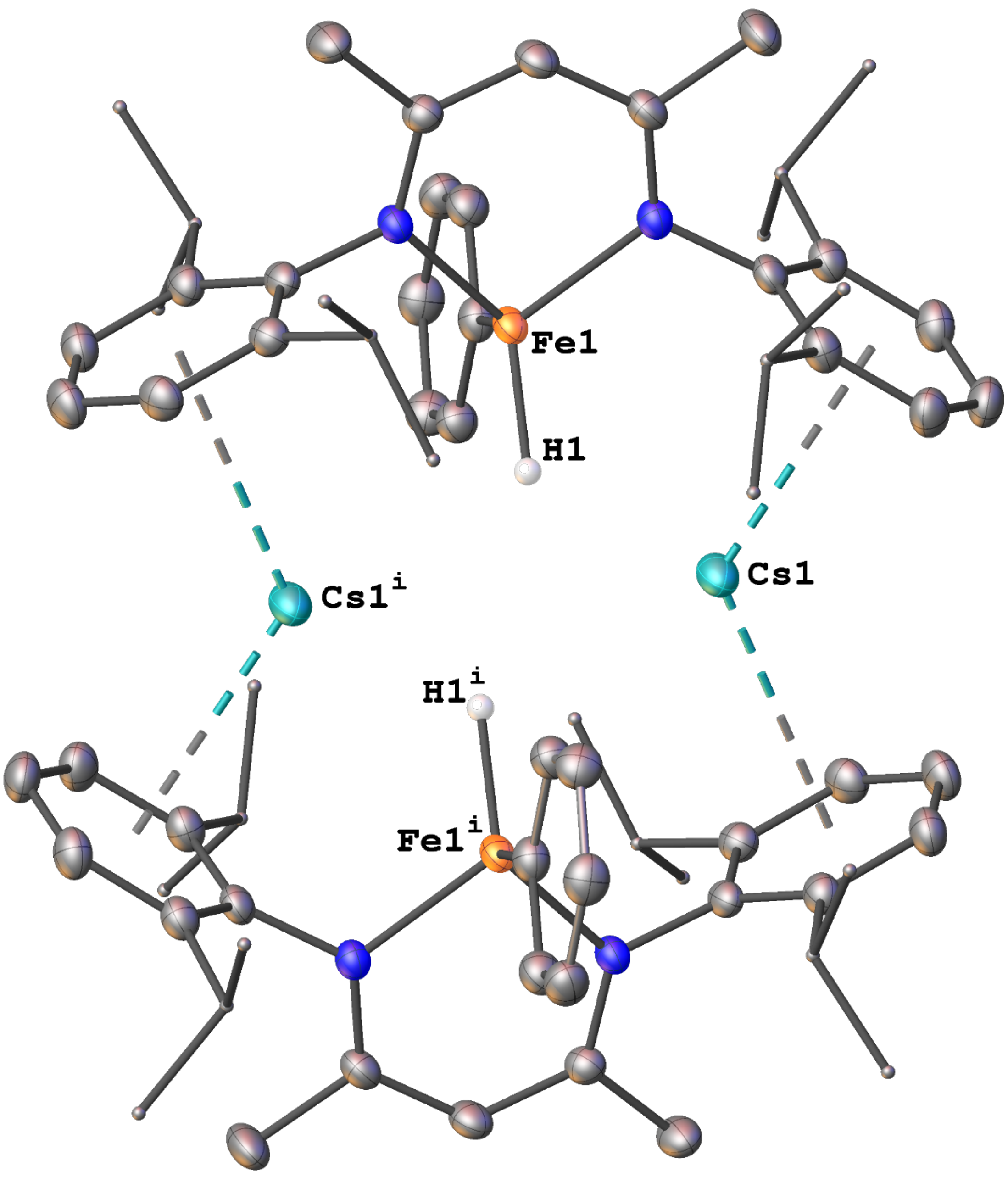

Figure S32. The complete dimer structure of syn-24026 with 50% thermal ellipsoid probability levels. The isopropyl fragments are shown as arbitrary sticks for clarity. All the hydrogen atoms are omitted, except for H1. Symmetry equivalent atoms are shown with superscript labels i that represent the symmetry operation $(1-x, 1-y, 1-z)$.

Table S3. X-ray crystal data and structure refinement for **3 (Cs)**.

Identification code	syn-24026	
Empirical formula	C70 H94 Cs2 Fe2 N4	
Formula weight	1369.01	
Temperature	105(2) K	
Wavelength	1.54184 Å	
Crystal system	Monoclinic	
Space group	$P2_1/n$	
Unit cell dimensions	a = 11.59130(10) Å	α= 90°.
	b = 18.90890(10) Å	β= 98.0400(10)°.
	c = 15.32260(10) Å	γ = 90°.
Volume	3325.38(4) $Å^3$	
Z	2	
Density (calculated)	1.367 g/cm^3	
Absorption coefficient	12.212 mm^{-1}	
F(000)	1408	
Crystal size	0.200 x 0.200 x 0.030 mm^3	
Crystal color and habit	red plate	
Diffractometer	XtaLAB Synergy, Dualflex, HyPix-Arc 100	
Theta range for data collection	3.735 to 74.498°.	
Index ranges	-14<=h<=14, -23<=k<=23, -19<=l<=19	
Reflections collected	100347	
Independent reflections	6804 [R(int) = 0.0456]	
Observed reflections (I > 2sigma(I))	6625	
Completeness to theta = 67.684°	100.0 %	
Absorption correction	Semi-empirical from equivalents	
Max. and min. transmission	1.00000 and 0.41153	
Solution method	SHELXT-2014/5 (Sheldrick, 2014)	
Refinement method	SHELXL-2014/7 (Sheldrick, 2014)	
Data / restraints / parameters	6804 / 0 / 366	
Goodness-of-fit on F^2	1.056	
Final R indices [I>2sigma(I)]	R1 = 0.0252, wR2 = 0.0604	
R indices (all data)	R1 = 0.0260, wR2 = 0.0610	
Largest diff. peak and hole	0.670 and -0.727 e.$Å^{-3}$	

Complex 3 (Cs) *NoSpherA2/HAR X-ray refinement*

The same data were refined against F^2 on all data by full-matrix least squares using Olex2-1.5, employing the NoSpherA2 implementation of Hirshfeld atom refinement (HAR).[2] Aspherical atomic scattering factors for all atoms were generated on-the-fly from Hirshfeld-partitioned electron densities calculated with ORCA at the DFT level using the PBE functional and the jorge-DZP-DKH basis set, including scalar relativistic effects via the DKH2 Hamiltonian for a neutral fragment (charge 0, multiplicity 9) with NoSpherA2 partitioning. All atoms, including hydrogen, were refined with anisotropic displacement parameters. The experimental data, structure refinement details are listed in Table S4. CCDC number 2514285 contains the detailed crystallographic data for this structure.

Complex 3 (Cs) *Neutron Diffraction*

Single-crystal neutron diffraction for **3 (Cs)$_{\mathbf{neutron}}$** was performed on TOPAZ at the Spallation Neutron Source, Oak Ridge National Laboratory, which uses a large-area array of Anger cameras to record neutron time-of-flight events in wavelength-resolved Laue mode.[3] A plate-like crystal (0.36 x 0.25 x 0.05 mm) was mounted on a MiTeGen loop with cyanoacrylate and measured at 100 K using a cryogen-free Cobra open-flow cooler (Oxford Cryosystems). The data-collection strategy was planned with NeuXtalViz;[4] Bragg peaks were integrated in three-dimensional HKL space using Mantid python program; and data reduction followed established TOPAZ protocols, including Lorentz, time-of-flight spectrum, and detector-efficiency corrections.[5] A spherical absorption correction was applied with a wavelength-resolved linear attenuation evaluated per reflection as $\mu(\lambda) = 0.14447 + 0.11103\ \lambda\ mm^{-1}$ (λ in Å). Reduced intensities were exported in SHELX HKLF2 Laue format with the per-reflection neutron wavelength retained.[6] The TOF-Laue wavelength band was 0.58–3.50 Å. For **3 (Cs)**, $C_{35}H_{47}CsFeN_2$, the formula contains 47 H per formula unit; with Z = 4 the unit cell contains 188 H atoms (about 54.7% of all atoms), consistent with elevated incoherent background and limited reflection statistics. Owing to the small crystal and extensive hydrogen content, relatively few peaks were recorded: 2128 reflections gave 874 unique (516 with $I > 2\sigma(I)$), R_{int} = 0.208, and limited completeness. This makes the neutron data highly sensitive to hydrogen but too sparse to support a full anisotropic model. We therefore used a targeted hybrid strategy. All non-hydrogen atoms (coordinates and ADPs) were taken from the prior NoSpherA2/HAR X-ray refinement and held fixed in the neutron refinement.[2] The hydrogen model is mixed: the Fe–H hydride H1 was located from neutron difference maps and refined with full anisotropic ADPs, whereas all other H atoms were placed in idealized positions (riding where appropriate) and refined with independent U_{iso} to convergence in SHELXL-2019/3.[7] The refinement used 34 parameters and 7 restraints for 874 reflections and converged to R1 = 0.097 for $I > 2\sigma(I)$ (R1(all) = 0.125), wR2 = 0.195 (obs)/0.208 (all), S = 0.93. The experimental data, structure refinement details are listed in Table S4. The coordination environment of the hydrides and atom labeling scheme is shown in Figure S33. CCDC number 2514457 contains the supplementary crystallographic data for the neutron structure.

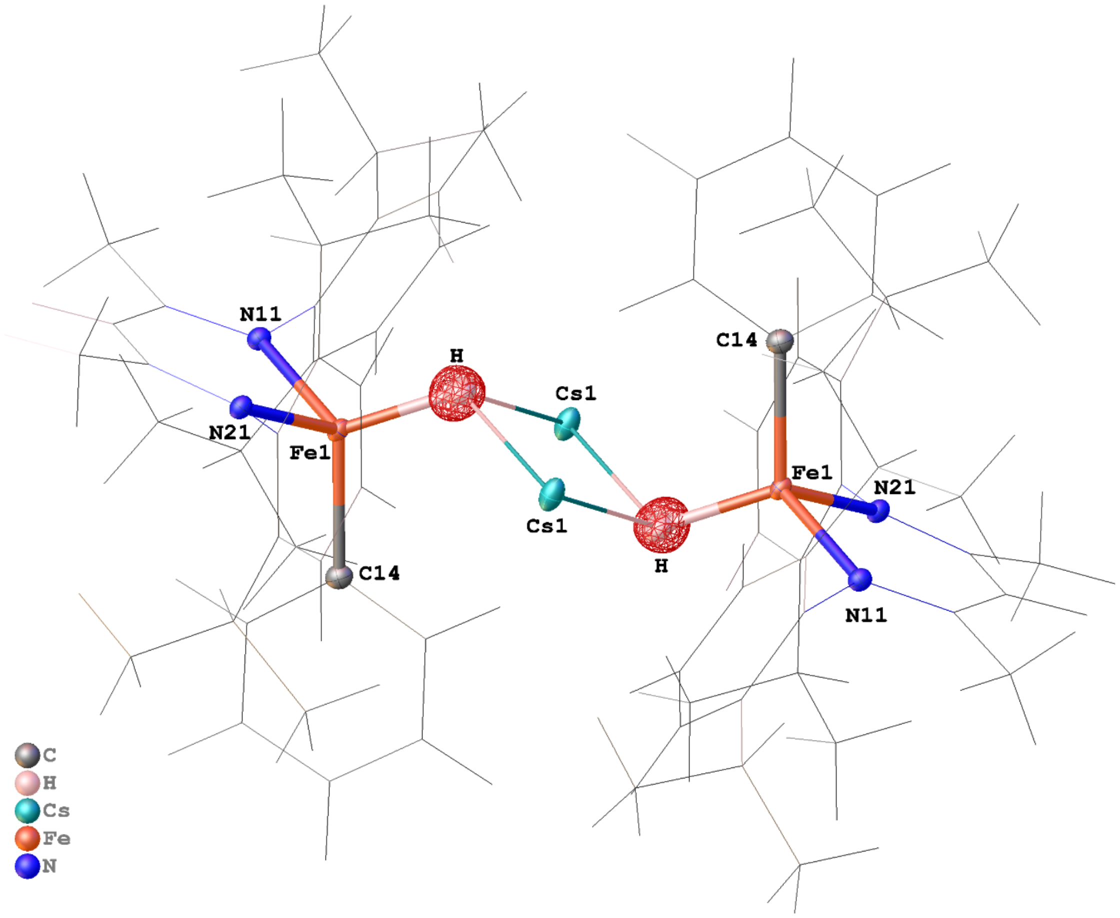

Figure S33. Neutron nuclear difference Fourier (omit) map for **3** (**Cs**). The bridging hydride H was omitted from the neutron model and an $F_o - F_c$ nuclear density map was calculated; the map is overlaid on the neutron-refined structure (30% probability ellipsoids). The mesh highlights the negative nuclear density (red) at the hydride site(s), consistent with ^{1}H (negative coherent scattering length), and confirms the hydride location between Fe1 and Cs1. Atoms of the ligand framework not directly bonded to Fe are shown as a wireframe for clarity. Contour level: $\pm 5\sigma$. Only negative contours are shown.

Table S4. Experimental Details for HAR and Neutron Refinement of **3 (Cs)**

	**Hirshfeld Atom Refinement**	**Neutron Structure**
Chemical formula	$C_{70}H_{94}Cs_2Fe_2N_4$	
M_r	1369.06	
Crystal system, space group	Monoclinic, $P2_1/n$	
Temperature (K)	105	100
a (Å)<br>b (Å)<br>c (Å)	11.5913 (1)<br>18.9089 (1)<br>15.3226 (1)	11.575(5)<br>18.883(8)<br>15.306(7)
β (°)	98.040 (1)	97.98(4)
V (Å^3)	3325.38 (4)	3313(3)
Z	2	2
Radiation type	Cu $K\alpha$	Neutrons, λ = 0.58 – 3.50 Å
μ (mm^{-1})	12.21	0.14447 + 0.11103 λ
Crystal size (mm)	0.2 × 0.2 × 0.03	0.25 × 0.36 × 0.05
Diffractometer	XtaLAB Synergy, Dualflex, HyPix-Arc 100	TOPAZ
Absorption correction	Multi-scan	Sphere
T_{min}, T_{max}	0.412, 1.000	0.803, 0.924
No. of measured,<br>Independent,<br>observed reflections	100347,<br>6804,<br>6625 [$I \geq 2\sigma(I)$]	2128,<br>874,<br>516 [$I > 2\sigma(I)$]
R_{int}	0.046	0.208
$(\sin \theta/\lambda)_{max}$ (Å^{-1})	0.625	0.973
$R[F^2 > 2\sigma(F^2)]$, $wR(F^2)$, S	0.021, 0.048, 1.08	0.097, 0.208, 0.93
No. of reflections	6804	874
No. of parameters	775	34
No. of restraints	0	7
H-atom treatment	All H-atom parameters refined	Hydride H atom refined
$\Delta\rho_{max}$, $\Delta\rho_{min}$	0.74, −0.64 (e Å^{-3})	0.77, −0.73 (fm Å^{-3})

Computer programs: SNS EPICS, TOPAZ Mantid Python Program, SHELXT 2014/5 (Sheldrick, 2014), olex2.refine 1.5 (Bourhis *et al.*, 2015), *SHELXL2019*/2 (Sheldrick, 2019), Olex2 1.5 (Dolomanov *et al.*, 2009). For direct comparison, the more accurate X ray lattice parameters were used in the neutron refinement.

Fe-K-ethylene complex

Low-temperature diffraction data (ω-scans) were collected on a Rigaku MicroMax-007HF diffractometer coupled to a Saturn994+ CCD detector with Cu Kα (λ = 1.54178 Å) for the structure of 007b-24050. The diffraction images were processed and scaled using Rigaku Oxford Diffraction software (CrysAlisPro; Rigaku OD: The Woodlands, TX, 2015). The structure was solved with SHELXT and was refined against F^2 on all data by full-matrix least squares with SHELXL (Sheldrick, G. M. Acta Cryst. 2008, A64, 112–122). All non-hydrogen atoms were refined anisotropically. Hydrogen atoms were included in the model at geometrically calculated positions and refined using a riding model. The isotropic displacement parameters of all hydrogen atoms were fixed to 1.2 times the U value of the atoms to which they are linked (1.5 times for methyl groups). The C–H hydrogen atoms bound to the iron-bound ethylene moieties could not be located for one of the two complexes in the asymmetric unit. The ethylene hydrogen atoms in the other complex were restrained using similar distance commands. The contribution of highly disordered tetrahydrofuran molecules in the lattice to the diffraction pattern was masked using the Olex2 implementation of SQUEEZE. The full numbering scheme of compound 007b-24050 can be found in the full details of the X-ray structure determination (CIF), which is included as Supporting Information. CCDC number 2516780 contains the detailed crystallographic data for this structure.

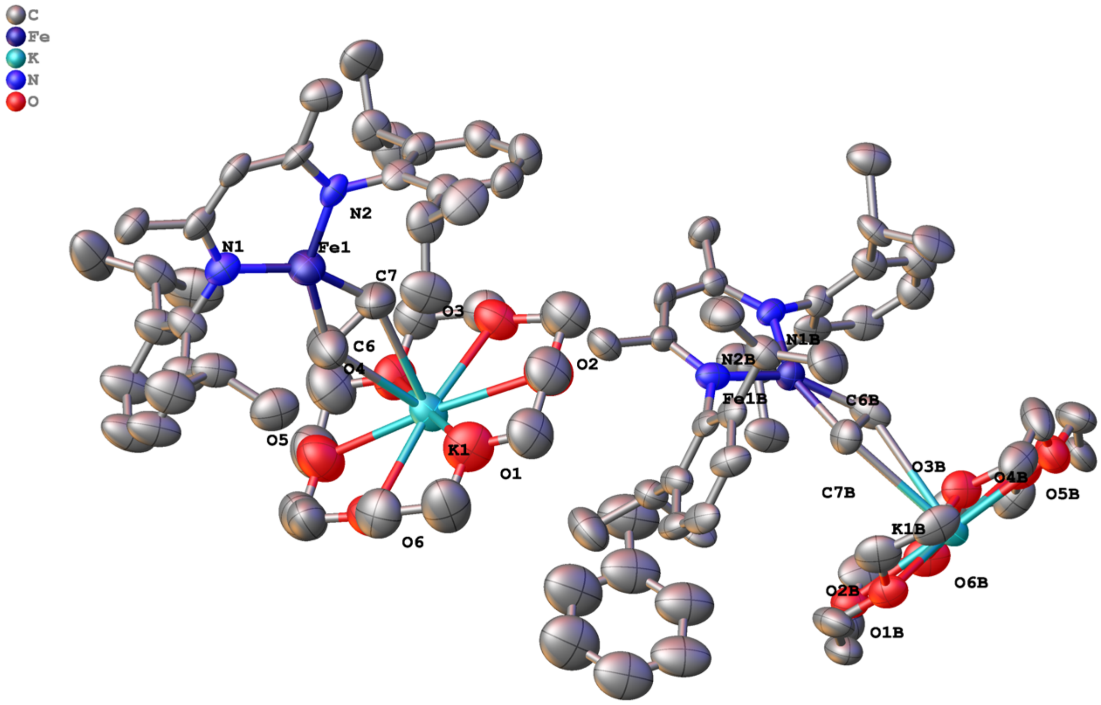

Figure S34. The complete numbering scheme of 007b-24050 with 50% thermal ellipsoid probability levels. The hydrogen atoms are omitted for clarity.

Table S5 Crystal data and structure refinement for **Fe-K-ethylene complex**.

Identification code	007b-24050	
Empirical formula	C46.50 H69 Fe K N2 O6	
Formula weight	846.98	
Temperature	100 K	
Wavelength	1.54184 Å	
Crystal system	Monoclinic	
Space group	$P2_1$	
Unit cell dimensions	a = 12.5751(3) Å	α= 90°.
	b = 17.5962(6) Å	β= 97.665(2)°.
	c = 21.7879(5) Å	γ = 90°.
Volume	4778.0(2) $Å^3$	
Z	4	
Density (calculated)	1.177 g/cm^3	
Absorption coefficient	3.666 mm^{-1}	
F(000)	1820	
Crystal size	0.2 x 0.2 x 0.2 mm^3	
Crystal color and habit	red block	
Diffractometer	XtaLAB AFC11 (RCD3): quarter-chi single	
Theta range for data collection	2.046 to 68.640°.	
Index ranges	-13<=h<=15, -14<=k<=21, -22<=l<=26	
Reflections collected	28002	
Independent reflections	14086 [R(int) = 0.1224]	
Observed reflections (I > 2sigma(I))	7563	
Completeness to theta = 67.684°	98.5 %	
Absorption correction	Semi-empirical from equivalents	
Max. and min. transmission	1.00000 and 0.25683	
Solution method	SHELXT 2018/2 (Sheldrick, 2018)	
Refinement method	SHELXL 2019/3 (Sheldrick, 2015)	
Data / restraints / parameters	14086 / 1391 / 1039	
Goodness-of-fit on F^2	1.082	
Final R indices [I>2sigma(I)]	R1 = 0.1097, wR2 = 0.2407	
R indices (all data)	R1 = 0.1918, wR2 = 0.2863	
Absolute structure parameter	-0.014(10)	
Largest diff. peak and hole	0.673 and -0.332 e.$Å^{-3}$	

Fe-Rb-ethylene complex

Low-temperature diffraction data (ω-scans) were collected on a Rigaku Synergy-S diffractometer coupled to a HyPix-Arc 100 detector with Cu Kα (λ = 1.54178 Å) for the structure of syn-24057. The diffraction images were processed and scaled using Rigaku Oxford Diffraction software (CrysAlisPro; Rigaku OD: The Woodlands, TX, 2015). The structure was solved with SHELXT and was refined against F^2 on all data by full-matrix least squares with SHELXL (Sheldrick, G. M. Acta Cryst. 2008, A64, 112–122). All non-hydrogen atoms were refined anisotropically. Hydrogen atoms were included in the model at geometrically calculated positions and refined using a riding model. The isotropic displacement parameters of all hydrogen atoms were fixed to 1.2 times the U value of the atoms to which they are linked (1.5 times for methyl groups). A disordered molecule of toluene was modeled over two positions using rigid bond restraints and similar thermal displacement parameter restraints. CCDC number 2516784 contains the detailed crystallographic data for this structure.

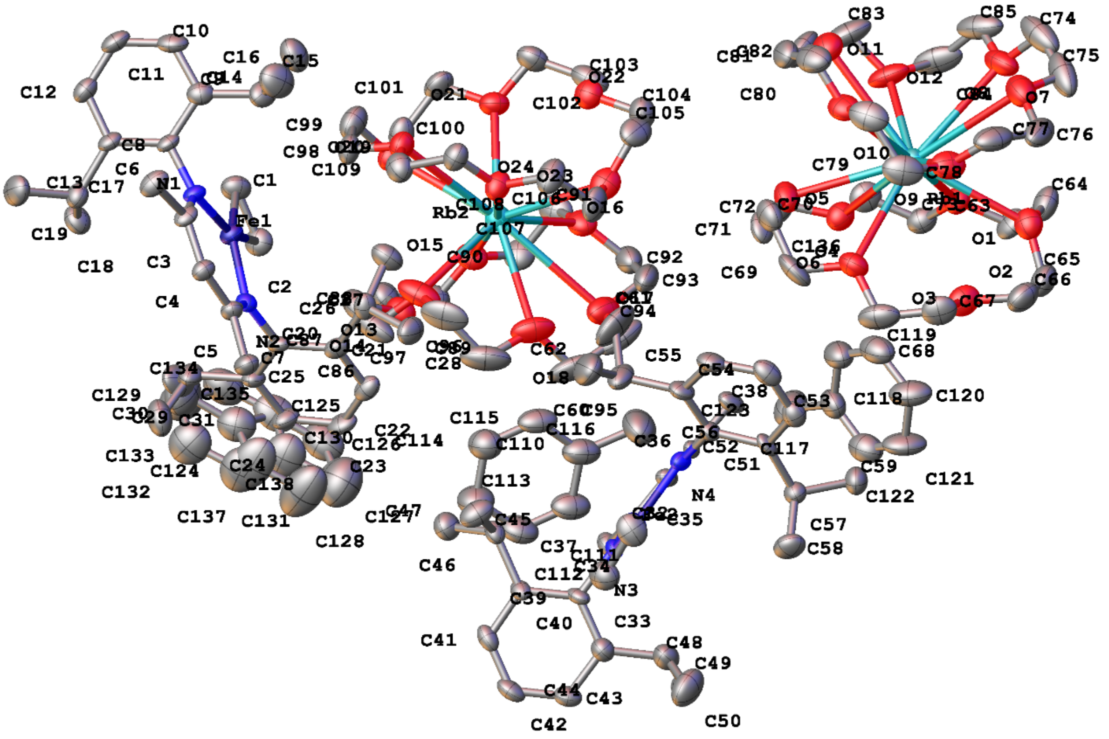

Figure S35. The complete numbering scheme of syn-24057 with 50% thermal ellipsoid probability levels. The hydrogen atoms are omitted for clarity.

Table S6. Crystal data and structure refinement for **Fe-Rb-ethylene complex**.

Identification code	syn-24057	
Empirical formula	C65.50 H105 Fe N2 O12 Rb	
Formula weight	1253.83	
Temperature	107.1(4) K	
Wavelength	1.54184 Å	
Crystal system	Orthorhombic	
Space group	$P2_12_12_1$	
Unit cell dimensions	a = 20.1945(2) Å	α= 90°.
	b = 25.8326(2) Å	β= 90°.
	c = 26.0611(2) Å	γ = 90°.
Volume	13595.5(2) Å^3	
Z	8	
Density (calculated)	1.225 g/cm^3	
Absorption coefficient	3.104 mm^{-1}	
F(000)	5368	
Crystal size	0.12 x 0.07 x 0.06 mm^3	
Crystal color and habit	red block	
Diffractometer	XtaLAB Synergy, Dualflex, HyPix-Arc 100	
Theta range for data collection	2.408 to 78.840°.	
Index ranges	-23<=h<=25, -32<=k<=31, -32<=l<=30	
Reflections collected	104902	
Independent reflections	28260 [R(int) = 0.0725]	
Observed reflections (I > 2sigma(I))	19670	
Completeness to theta = 67.684°	99.6 %	
Absorption correction	Semi-empirical from equivalents	
Max. and min. transmission	1.00000 and 0.47679	
Solution method	SHELXT 2018/2 (Sheldrick, 2018)	
Refinement method	SHELXL 2019/3 (Sheldrick, 2015)	
Data / restraints / parameters	28260 / 528 / 1526	
Goodness-of-fit on F^2	1.121	
Final R indices [I>2sigma(I)]	R1 = 0.0858, wR2 = 0.1611	
R indices (all data)	R1 = 0.1268, wR2 = 0.1812	
Absolute structure parameter	0.047(7)	
Largest diff. peak and hole	0.838 and -0.791 e.Å^{-3}	

[Cs(18-crown-6)$_2$][LFe(Ph)(H)]

Low-temperature diffraction data (ω-scans) were collected on a Rigaku Synergy-S diffractometer coupled to a HyPix-Arc 100 detector with Cu Kα (λ = 1.54178 Å) for the structure of syn-24060. The diffraction images were processed and scaled using Rigaku Oxford Diffraction software (CrysAlisPro; Rigaku OD: The Woodlands, TX, 2015). The structure was solved with SHELXT and was refined against F^2 on all data by full-matrix least squares with SHELXL (Sheldrick, G. M. Acta Cryst. 2008, A64, 112–122). All non-hydrogen atoms were refined anisotropically. Hydrogen atoms were included in the model at geometrically calculated positions and refined using a riding model, except the hydride which was located and refined with an isotropic thermal parameter. The isotropic displacement parameters of all carbon-bound hydrogen atoms were fixed to 1.2 times the U value of the atoms to which they are linked (1.5 times for methyl groups).

The two cesium crown ether complexes present in the asymmetric unit were extensively disordered, and despite the usage of numerous restraints (such as similar thermal displacement parameter restraints, similar distance restraints, rigid bond restraints, and isotropic displacement parameter restraints) these disordered complexes could not be modeled entirely. Attempts to simply mask away the complexes resulted in total degradation of the entire model. Therefore, the partially modeled complexes have been included for completeness, but bond metrics associated with these atoms will not be reliable. The iron complex itself is not significantly disordered, and the hydride atom was located from the Fourier difference map.

The contribution of highly disordered toluene to the diffraction pattern was masked using the OLEX2 implementation of SQUEEZE (*Acta Crystallogr. D* **2005**, *61*, 1299–1301). CCDC number 2516785 contains the detailed crystallographic data for this structure.

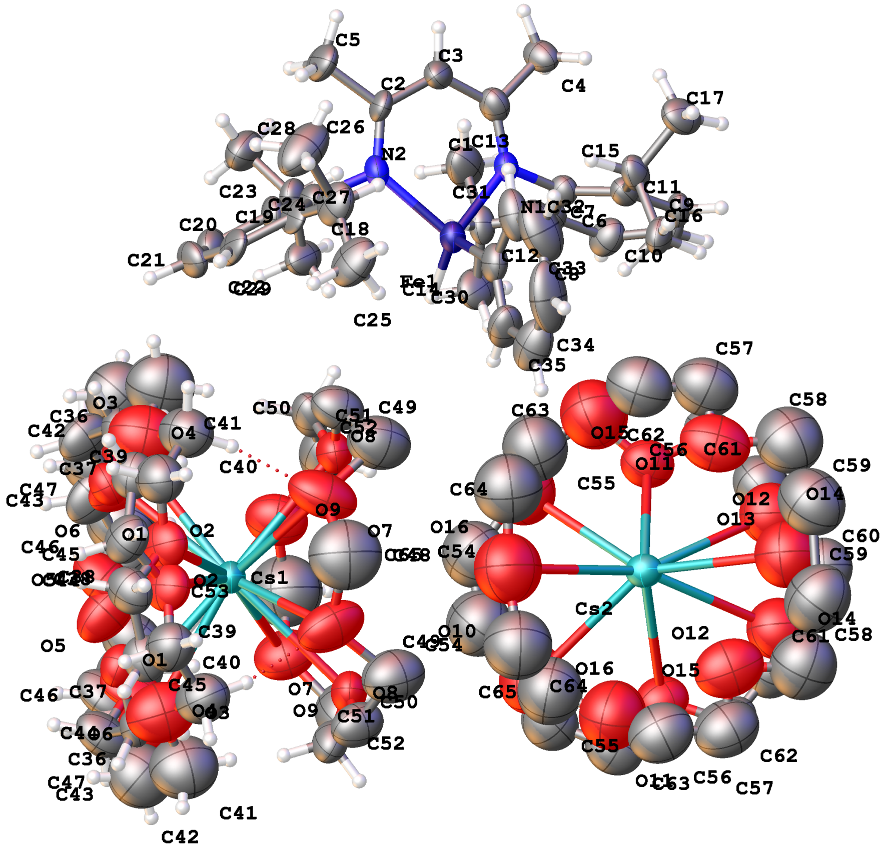

Figure S36. The complete numbering scheme of $[Cs(18\text{-crown-}6)_2][LFe(Ph)(H)]$ with 50% thermal ellipsoid probability levels. The hydrogen atoms are shown as circles.

Table S7. Crystal data and structure refinement for **[Cs(18-crown-6)$_2$][LFe(Ph)(H)]**.

Identification code	syn-24060	
Empirical formula	C71.50 H80 Cs Fe N2 O12	
Formula weight	1348.13	
Temperature	107.2(6) K	
Wavelength	1.54184 Å	
Crystal system	Orthorhombic	
Space group	Pnma	
Unit cell dimensions	a = 23.9088(4) Å	α= 90°.
	b = 26.2643(5) Å	β= 90°.
	c = 25.6126(5) Å	γ = 90°.
Volume	16083.4(5) Å^3	
Z	8	
Density (calculated)	1.114 g/cm^3	
Absorption coefficient	5.396 mm^{-1}	
F(000)	5600	
Crystal size	0.22 x 0.13 x 0.13 mm^3	
Crystal color and habit	red block	
Diffractometer	XtaLAB Synergy, Dualflex, HyPix-Arc 100	
Theta range for data collection	2.410 to 78.775°.	
Index ranges	-30<=h<=28, -30<=k<=31, -30<=l<=29	
Reflections collected	271846	
Independent reflections	15758 [R(int) = 0.1596]	
Observed reflections (I > 2sigma(I))	12569	
Completeness to theta = 67.684°	100.0 %	
Absorption correction	Semi-empirical from equivalents	
Max. and min. transmission	1.00000 and 0.20238	
Solution method	SHELXT 2018/2 (Sheldrick, 2018)	
Refinement method	SHELXL 2019/3 (Sheldrick, 2015)	
Data / restraints / parameters	15758 / 883 / 720	
Goodness-of-fit on F^2	1.680	
Final R indices [I>2sigma(I)]	R1 = 0.1408, wR2 = 0.4000	
R indices (all data)	R1 = 0.1588, wR2 = 0.4149	
Extinction coefficient	n/a	
Largest diff. peak and hole	2.246 and -1.458 e.Å^{-3}	

Alternative Fits for Mössbauer Spectra

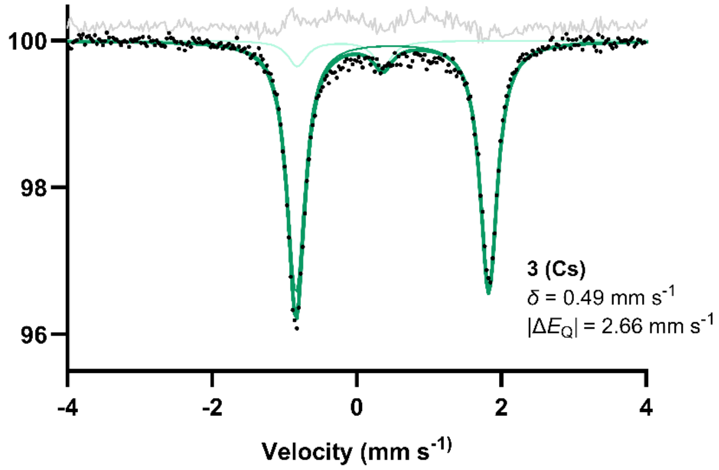

Figure S37. Alternative fit of **3** as a major component, with a 10% impurity having isomer shift -0.23 mm/s and quadrupole splitting 1.18 mm/s. We deem this fit to be unlikely to be correct because of the unusual isomer shift.

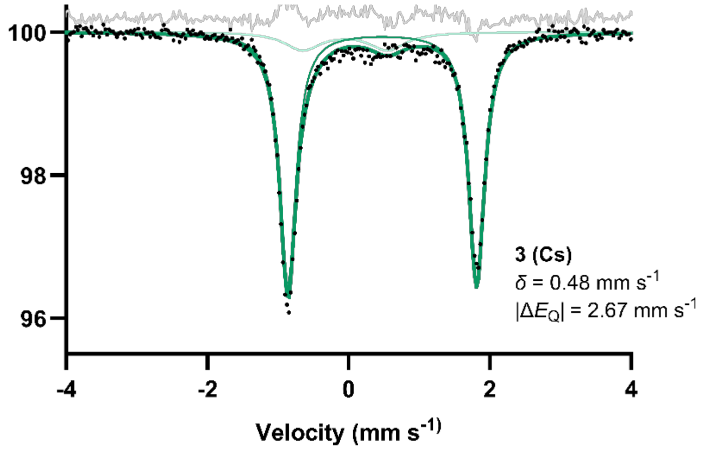

Figure S38. Alternative fit of **3** as a major component, with a 13% impurity having isomer shift -0.05 mm/s and quadrupole splitting 1.20 mm/s. This fit required constraining the isomer shift of the major component.

References for Supporting Information

(1) Smith, J. M. Studies of Low-Coordinate Iron Dinitrogen Complexes. *J. Am. Chem. Soc.* **2006**, *128*, 756–769.

(2) Kleemiss, F.; Dolomanov, O. V.; Bodensteiner, M.; Peyerimhoff, N.; Midgley, L.; Bourhis, L. J.; Genoni, A.; Malaspina, L. A.; Jayatilaka, D.; Spencer, J. L.; et al. Accurate crystal structures and chemical properties from NoSpherA2. *Chem. Sci.* **2021**, *12*, 1675-1692.

(3) Coates, L.; Cao, H. B.; Chakoumakos, B. C.; Frontzek, M. D.; Hoffmann, C.; Kovalevsky, A. Y.; Liu, Y.; Meilleur, F.; dos Santos, A. M.; Myles, D. A. A.; et al. A suite-level review of the neutron single-crystal diffraction instruments at Oak Ridge National Laboratory. *Rev. Sci. Instr.* **2018**, *89*, 092802.

(4) Oak Ridge National Laboratory, *NeuXtalViz*, https://single-crystal.ornl.gov/software/neuxtalviz/index.html (accessed Dec. 19, 2025).

(5) Schultz, A. J.; Jorgensen, M. R. V.; Wang, X. P.; Mikkelson, R. L.; Mikkelson, D. J.; Lynch, V. E.; Peterson, P. F.; Green, M. L.; Hoffmann, C. M. Integration of neutron time-of-flight single-crystal Bragg peaks in reciprocal space. *J. Appl. Cryst.* **2014**, *47*, 915-921. DOI: 10.1107/s1600576714006372.

(6) Sheldrick, G. M. Crystal structure refinement with *SHELXL*. *Acta Cryst. C* **2015**, *71*, 3-8. DOI: 10.1107/s2053229614024218.
